# Microbial Natural Products Targeting the *Mycobacterium tuberculosis* Complex: Toward the Discovery of Novel Anti-Tubercular Agents

**DOI:** 10.3390/microorganisms14091933

**Published:** 2026-09-01

**Authors:** Veronica Folliero, Federica Dell’Annunziata, Giulia Radocchia, Raimondo Leone, Maria Rosaria Gualano, Alessandra Fusco, Lorenza Putignani

**Affiliations:** 1 Department of Life Science, Health, and Health Professions, Link Campus University, 00165 Rome, Italy; f.dellannunziata@unilink.it (F.D.); g.radocchia@unilink.it (G.R.); alessandra.fusco@unilink.it (A.F.); 2 Section of Hygiene, Department of Life Sciences and Public Health, Università Cattolica del Sacro Cuore, 00168 Rome, Italy; raimondo.leone@unicatt.it; 3 Saint Camillus International University of Health and Medical Sciences, 00131 Rome, Italy; mariarosaria.gualano@unicatt.it; 4 Leadership in Medicine Research Center, Università Cattolica del Sacro Cuore, 00168 Rome, Italy; 5 Unit of Microbiomics and Unit of Research of Microbiome, Bambino Gesù Children’s Hospital, IRCCS, 00165 Rome, Italy

**Keywords:** tuberculosis, mycobacteria, microbial natural products, antimycobacterial activity, anti-tubercular agents

## Abstract

Tuberculosis (TB) remains one of the most significant global public health challenges, as it represents the world’s leading infectious cause of death. The clinical efficacy of currently available anti-TB drugs is increasingly compromised due to the growing prevalence of antibiotic-resistant strains, side effects, and prolonged treatment times. This scenario highlights the urgent need to identify new anti-TB drugs with alternative mechanisms of action and improved anti-TB activity and bioavailability. In this context, natural products derived from microorganisms represent a key source of chemical diversity for drug discovery. Actinomycetes, fungi, and other environmental microbes produce a wide range of secondary metabolites with broad antimicrobial activity. These compounds can interfere with essential bacterial structures and processes, including membrane integrity, redox homeostasis, protein synthesis, and DNA replication. This review aims to evaluate new microbial natural products for their antibacterial activity, focusing on their efficacy, mechanisms of action, chemical nature, and potential clinical applications, with the aim of informing the development of new anti-TB agents.

## 1. Introduction

Tuberculosis (TB) is currently one of the top 10 infectious causes of death worldwide. In 1993, the World Health Organization (WHO) declared TB a global health crisis [1]. Unfortunately, even after more than three decades, this infection still poses a serious threat. According to the WHO Global Tuberculosis Report 2025, approximately 10.7 million people developed TB, of whom 1.23 million died from the infection. Globally, the TB incidence is estimated at 131 cases per 100,000 population, with a case fatality rate of 11.5%. Approximately 87% of global TB cases were reported in 30 countries, including India (25%), Indonesia (10%), the Philippines (6.8%), China (6.5%), Pakistan (6.3%), Nigeria (4.8%), the Democratic Republic of the Congo (3.9%), and Bangladesh (3.6%) [2]. After three years of rising cases due to healthcare disruptions caused by the COVID-19 pandemic, the number of cases has seen a slight decline. Specifically, incidence rates decreased by 1.7%, returning to levels observed in 2020 [3]. However, these advances are insufficient to meet the targets proposed by the WHO. In fact, from 2015 to 2024, the incidence reduction was only 12%, not 50% as expected [4].

TB is caused by the *Mycobacterium tuberculosis* complex (MTBC), which includes the following Mycobacterium species: *Mycobacterium africanum* (*M. africanum*), *Mycobacterium bovis* (*M. bovis*), *Mycobacterium cannetti* (*M. canettii*), *Mycobacterium caprae* (*M. caprae*), *Mycobacterium microti* (*M. microti*), *Mycobacterium mungi* (*M. mungi*), *Mycobacterium orygis* (*M. orygis*), *Mycobacterium pinnipedii* (*M. pinnipedii*), *Mycobacterium suricattae* (*M. suricattae*) and *Mycobacterium tuberculosis* (*M. tuberculosis*) [5]. Despite sharing more than 99% genetic homology, they exhibit significant variability in their biochemical profiles, pathogenicity, and the infected hosts. Regarding the latter aspect, MTBC includes zoonotic species and strains exclusively adapted to humans or animals. Specifically, *M. tuberculosis*, *M. africanum*, and *M. canettii* primarily affect humans. *M. bovis*, *M. caprae*, and *M. orygis* have zoonotic potential, while *M. mungi* and *M. suricattae* mainly infect animal hosts [6].

Current drug therapy for TB is severely hampered by lengthy treatment durations, side effects, and the emergence of antibiotic-resistant strains. Drug-susceptible tuberculosis (DS-TB), defined as sensitivity to isoniazid and rifampin, is traditionally treated with a 6-month regimen of isoniazid, rifampin, pyrazinamide, and ethambutol, although shorter regimens are now available for selected patients [7]. Drug-resistant tuberculosis (DR-TB) includes isoniazid-resistant (Hr-TB), rifampin-resistant (RR-TB), multidrug-resistant (MDR-TB), pre-extensively drug-resistant (pre-XDR-TB), and extensively drug-resistant (XDR-TB) TB. Treatment is tailored based on resistance profile and has progressively shifted toward shorter, all-oral regimens incorporating newer agents such as bedaquiline, pretomanid, linezolid, delamanid, and clofazimine [8]. Current WHO recommendations include 6-month regimens for many patients with MDR/RR-TB, while longer, individualized regimens remain necessary for patients with more extensive drug resistance [9]. Side effects of anti-tuberculosis drugs are common with both first- and second-line drugs, with more varied and severe reactions in the latter. They generally include gastrointestinal problems, pancreatitis, hepatitis, rash, and fever [10].

Globally, 390,000 cases were reported in 2024. Approximately two-thirds of global TB cases occurred in India (25%), Indonesia (10%), the Philippines (6.8%), China (6.5%), Pakistan (6.3%), Nigeria (4.8%), the Democratic Republic of the Congo (3.9%), and Bangladesh (3.6%) [11]. Although TB remains one of the most prevalent and globally impactful mycobacterial diseases, infections caused by nontuberculous mycobacteria (NTM) should not be underestimated and represent an emerging therapeutic challenge. NTM comprises over 150 species, including clinically relevant pathogens such as *Mycobacterium avium*, *Mycobacterium kansasii*, and *Mycobacterium abscessus*, which cause pulmonary and extrapulmonary infections. These microorganisms exhibit intrinsic antimicrobial resistance, sharing several mechanisms of resistance with MTBC members, including reduced drug permeability due to the complex mycobacterial cell envelope, enzymatic drug modification, target alteration, and active efflux pumps. However, given the global disease burden, widespread clinical impact, and the increasing emergence of drug-resistant MTBC strains, this review focuses primarily on tuberculosis caused by MTBC.

In view of these challenges, the detection of innovative treatments with alternative mechanisms of action, shorter treatment durations, and efficacy against resistant strains becomes crucial [12].

Natural products produced by microorganisms constitute an important reservoir of biologically active compounds. They exhibit a wide variety of pharmacological properties, including antimicrobial, antitumor, and immunomodulatory activity [13]. Over the last century, microbial natural products have contributed significantly to the discovery and development of drugs for the treatment of infectious diseases. A well-known example is streptomycin, one of the first antibiotics used in TB treatment. It is an aminoglycoside antibiotic originally isolated from the soil actinomycete *Streptomyces griseus* (*S. griseus*) as a secondary metabolite. Its discovery represented a breakthrough in antimicrobial treatment and clearly highlighted the essential role of microbial-derived natural products in the development of anti-TB drugs [14].

Given the continuing public health threat posed by tuberculosis, including the rise in multidrug-resistant strains, this review examines microbial natural products with demonstrated activity against the MTBC. We describe, for each compound, its chemical characteristics, antitubercular potency, underlying mechanisms of action, and clinical translatability. This synthesis is intended to serve as a foundation for the rational design and development of next-generation anti-TB agents, in support of global tuberculosis control efforts.

## 2. Targets of *M. tuberculosis* for Drug Discovery

### 2.1. Cell-Wall Biosynthesis

The highly complex architecture of the MTBC cell wall plays a central role in virulence, intrinsic antibiotic resistance, and host–pathogen interactions. The mycoyl–arabinogalactan–peptidoglycan (mAGP) complex constitutes the core structure of the MTBC cell wall [15].

**Figure 1:**
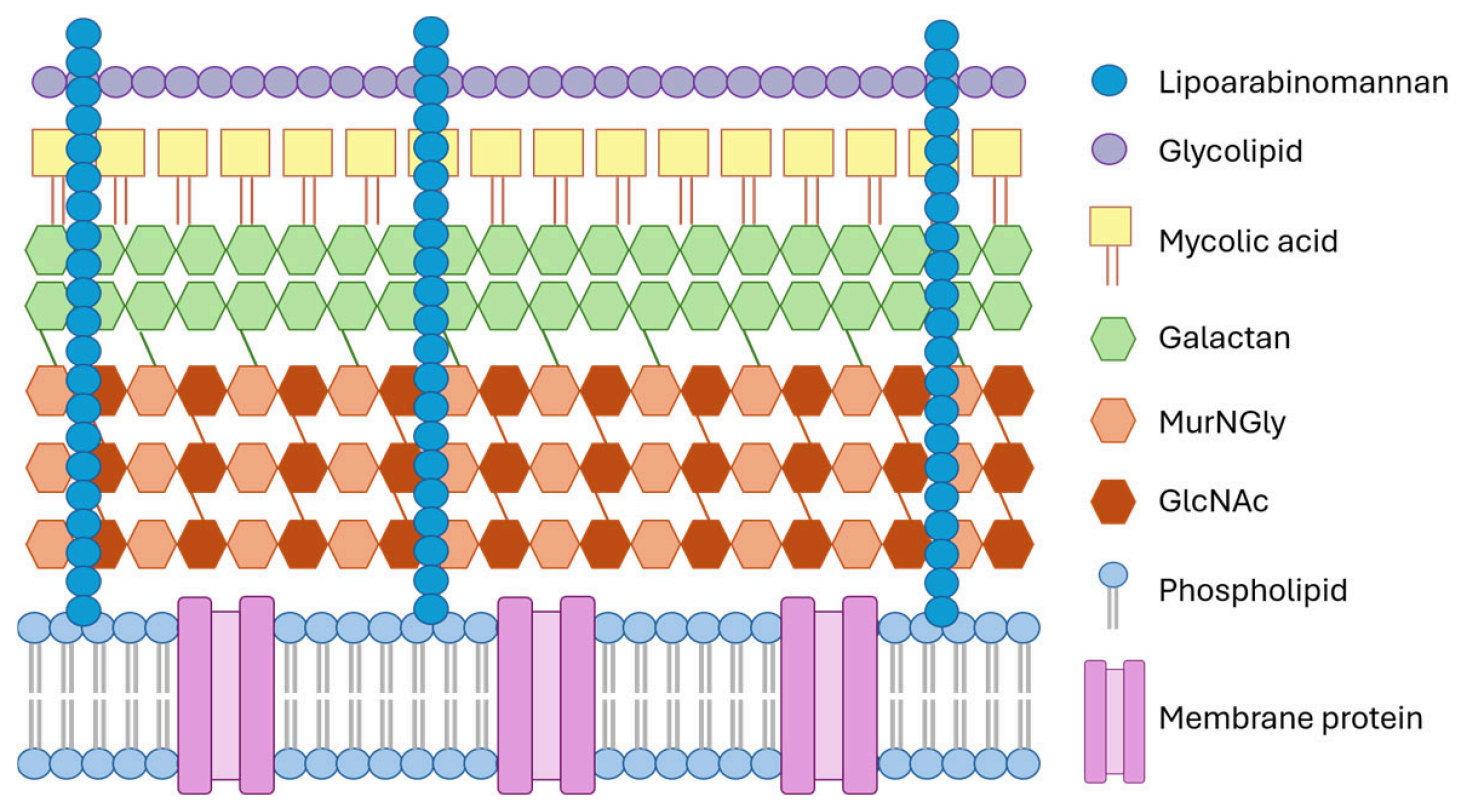
Schematic representation of mycobacterial cell envelope, characterized by peptidoglycan covalently linked to arabinogalactan, which in turn is esterified with mycolic acids. Lipoarabinomannans are present, which serve as linking structures. Glycolipids, involved in virulence and host interaction, are also present on the external surface.

Peptidoglycan (PG) forms the basal layer of the mAGP and lies outside the plasma membrane. It is essential for maintaining the cell’s shape and structural integrity and provides resistance to external osmotic pressure. PG is formed by the succession of N-acetylglucosamine (GlcNAc) and N-acetylmuramic acid (MurNAc), linked by a β1 → 4 bond [16]. Arabinogalactan (AG) is a heteropolysaccharide that links peptidoglycan (PG) to external mycolic acids (MAs). AG is a heteropolysaccharide that links PG to external MAs. It consists of three main domains: linker, core, and outer unit. The core contains galactose chains, while the outer domain is formed by arabinan, a highly branched structure consisting of arabinofuranose (Araf) residues linked mainly by α(1 → 5) bonds, with α(1 → 3) branches [15]. The outer layer of the cell wall is composed of MAs, covalently linked to GA. They consist of very-long-chain α-alkylated and β-hydroxylated fatty acids, typically between 60 and 90 carbon atoms. Their structural variations, including oxygenated and non-oxygenated forms, enhance resistance to acid-alcohols and limit the penetration of conventional antibiotics [17]. The cell envelope also contains glycolipids and lipoglycans, including lipoarabinomannan (LAM), anchored to the plasma membrane via phosphatidylinositol, and phosphatidylinositol mannosides (PIM), which contribute to cell-wall organization, growth, virulence, immune evasion, and modulation of host responses [18] (Figure 1).

Due to the enormous complexity of the MTBC cell-wall structure, several antimicrobials have been developed to block its assembly or the synthesis of precursors. Therefore, AG, LAM and MAs represent specific and major anti-TB targets [19]. AG and LAM biosynthesis is mediated by arabinosyltransferases encoded by the emb (embA, embB, embC) and aft (aftA–D) gene clusters. EmbA/B primarily catalyze AG elongation, whereas EmbC contributes to LAM biosynthesis. Aft enzymes sequentially mediate arabinan initiation, branching, extension, and the addition of terminal Araf residues required for MA attachment [20,21]. Ethambutol (EMB), the only clinically used drug acting on this pathway, inhibits the EmbABC complex, disrupting AG/LAM assembly, reducing MA attachment sites, and compromising cell-wall integrity [22]. EMB is used for active TB at 15–25 mg/kg/day in combination therapy [23]. MA biosynthesis involves two fatty acid synthase systems: FAS-I, which generates fatty acid precursors, and FAS-II, which elongates them. Within FAS-II, the NADH-dependent enoyl-ACP reductase InhA catalyzes a key step in the formation of meromycolic acids, which are subsequently incorporated into mature MAs [24]. Isoniazid (INH) and ethionamide (ETH) inhibit InhA following activation by the mycobacterial enzymes KatG and EthA, respectively, thereby disrupting MA biosynthesis [25,26]. INH is used for active and latent tuberculosis at doses of 5–15 mg/kg/day, whereas ETH is used primarily for active drug-resistant tuberculosis at doses of 15–20 mg/kg/day, both in combination with other antituberculosis drugs [25,26,27].

### 2.2. Targeting Nucleic Acid

The MTBC genome possesses remarkable characteristics that explain its high resistance to environmental and host factors, as well as its stable evolution over time. It is approximately 4.4 Mb in size, with a high GC content (~65%), which contributes to adaptation to extreme conditions [28]. The MTBC genome is highly conserved and primarily evolves through clonal reproduction, with minimal horizontal gene transfer. Therefore, strain diversity mainly arises from mutations accumulated through DNA replication errors, intracellular oxidative stress, and incomplete DNA repair [29]. It contains approximately 4000 genes, including 3900–3950 protein-coding genes involved in essential processes such as intermediary metabolism, DNA replication and repair, cell-wall biosynthesis, virulence, and adaptation to host and environmental stresses [30]. The remaining genes include approximately 45–50 tRNA genes, 3–4 rRNA operons, and numerous small non-coding RNAs (sRNAs) involved in gene regulation in response to stress [31]. Despite their essential roles, nucleic acid biosynthesis and associated cellular processes remain largely unexplored as therapeutic targets, representing promising opportunities for antituberculosis drug discovery.

#### 2.2.1. DNA Replication

DNA replication in MTBC is mediated by a multiprotein replisome that includes DNA polymerase III, DnaB helicase, DnaG primase, the β clamp, and the clamp-loading complex [32]. MTBC possesses simplified replication machinery compared to Escherichia coli (*E. coli*), lacking several accessory components while retaining functions necessary for chromosome duplication [33]. Replication is initiated by the binding of DnaA-ATP to OriC, followed by DnaB loading and DNA strand separation; notably, MTBC lacks the DnaC helicase loader present in *E. coli* [33]. Its replisome also lacks the χ, ψ, γ, and θ subunits, with the absence of θ possibly affecting replication fidelity [34,35,36,37,38]. Despite the essentiality of these components, no currently approved anti-tuberculosis drug directly targets the replisome or DNA polymerase machinery, highlighting this pathway as a potential target for drug discovery [39].

DNA supercoiling is regulated by topoisomerases that maintain DNA conformation during replication and transcription. In *M. tuberculosis*, TopA is the only type I topoisomerase, while DNA gyrase is the only type II topoisomerase and is an established target of fluoroquinolones [40]. DNA gyrase is composed of the subunits GyrA and GyrB: GyrA binds to DNA and mediates strand cleavage/religation, while GyrB provides the ATPase activity required for catalysis [41]. Fluoroquinolones, including levofloxacin, moxifloxacin, ofloxacin, and gatifloxacin, target GyrA, stabilizing DNA cleavage intermediates and causing the accumulation of double-strand breaks [10]. The recommended doses are 400 mg/day for moxifloxacin, 10–15 mg/kg/day for levofloxacin, 400 mg/day for gatifloxacin and 400 mg twice daily for ofloxacin, administered orally or, if appropriate, intravenously in combination regimens [42].

#### 2.2.2. DNA Repair

Continuous DNA damage induced by reactive oxygen and nitrogen species, coupled with the absence of the θ subunit involved in stabilizing polymerase proofreading, threatens the stability of the MTBC genome [43]. There are three major repair pathways, including mismatch repair (MMR), base excision repair (BER), and nucleotide excision repair (NER) [44]. The NucS-dependent MMR system corrects post-replicative mismatches through β-clamp-mediated recognition and incision, followed by DNA resynthesis and ligation. NucS deficiency markedly increases spontaneous mutation rates [45,46,47]. BER repairs oxidized, deaminated, and alkylated bases through DNA glycosylase, AP endonucleases, DNA polymerase, and ligase. Instead, NER removes bulky DNA lesions via the UvrABC complex, followed by UvrD-mediated excision, resynthesis, and ligation [48,49]. Despite their importance for genome stability, DNA repair systems are not currently targeted by approved anti-TB drugs and therefore represent promising targets for novel therapeutic strategies.

#### 2.2.3. DNA Transcription

Bacterial RNA polymerase is the central enzyme of transcription. The enzymatic core of RNA polymerase is composed of the α_2_ββ′ω subunits. The α subunits mediate enzyme assembly and transcription regulation. The β′ and β subunits form the catalytic center, responsible for binding to DNA and incorporating ribonucleotides into the nascent RNA strand. Finally, the ω subunit stabilizes the enzyme complex, contributing to its functional stability [50]. RNA polymerase recognizes specific promoters owing to its bound sigma factor. During active growth, the enzyme binds to σA. It recognizes the −35 and −10 regions of the promoter via the σ4 and σ2 domains, respectively. These interactions are essential for the local unwinding of the DNA double helix and the formation of the open transcription complex (RPo) [51]. When the bacterial complex experiences stress, other sigma factors come into play, reprogramming gene expression to favor latency rather than growth, including SigH, SigE and others [52]. Among anti-TB drugs, rifampicin affects the transcriptional process. This first-line drug interacts with the β-subunit of RNA polymerase, inhibiting mRNA synthesis [53]. Rifampicin is administered orally, and in selected cases intravenously, at a daily dose of 10–20 mg/kg as part of combination regimens for the treatment of active TB and selected cases of latent TB infection [54] (Table 1).

### 2.3. Inhibition of Protein Synthesis

Protein synthesis occurs on the ribosome, the target of nearly 40% of conventional clinical antibiotics. It consists of: (i) a small subunit responsible for decoding mRNA; and (ii) a large subunit that guides the peptide bond between the nascent polypeptide and the new amino acid. Both subunits form a functional ribosome with a sedimentation coefficient of 70S [55]. The 70S ribosome contains three tRNA-binding sites: the aminoacyl (A) site, the peptidyl (P) site, and the exit (E) site. MTBC ribosomes retain the canonical bacterial architecture, with a large 50S subunit containing 37 proteins and 23S/5S rRNA and a small 30S subunit containing 21 proteins and 16S rRNA [56]. However, they display distinctive structural features, including an extended 23S rRNA helix that forms the B9 bridge with bS6, which increases stability during intracellular latency, and additional RNA loops involved in translation regulation [57,58]. MTBC also lacks bS21, favoring the translation of leaderless mRNAs, while specific proteins bL37 and bS22 contribute to translation efficiency and modify interactions with antibiotics [56,59]. Under conditions of nutrient and zinc limitation, ribosomes can go into hibernation, which involves the replacement of C+ proteins with C− and MPY-mediated blockade of the A and P sites, thus protecting ribosomes from degradation and drug activity. A subset of ribosomes remains active for the translation of latency-associated genes [60] (Table 2).

The main anti-TB drugs that affect protein synthesis include streptomycin (STR) and amikacin, both aminoglycosides. STR binds to the 30S subunit via the 16S and S12 rRNA, inhibiting translation initiation [61]. Otherwise, amikacin binds to the A site, altering its structure, disrupting mRNA-tRNA base pairing, and causing mistranslation [62]. STR is administered intramuscularly at a dose of 15 mg/kg once daily (maximum 1 g/day), while amikacin is administered intravenously at a dose of 15–20 mg/kg once daily for active tuberculosis [63].

### 2.4. Protein Quality-Control System

Proteostasis in bacteria is a tightly regulated and dynamic process, controlled by the balance between protein synthesis, proper folding, and degradation. This process is largely driven by environmental signals. A pathogen’s ability to perceive and respond to these stimuli is a key determinant of its adaptability and virulence [64].

The bacterial protein quality control (PQC) system consists of molecular chaperones and ATP-dependent proteases, which work together to maintain the correct conformation of cellular proteins and eliminate damaged or misfolded proteins. Specifically, molecular chaperones promote the correct folding or refolding of unfolded and misfolded proteins. Proteases, on the other hand, degrade proteins irreversibly, allowing for the recovery and reuse of individual amino acids [65]. In mycobacteria, the DnaK (Hsp70) system, together with the co-chaperones DnaJ and GrpE, is central to PQC. DnaJ recognizes and delivers misfolded proteins to DnaK, while GrpE promotes ADP release and ATP binding, enabling subsequent chaperone cycles [66].

**Figure 2:**
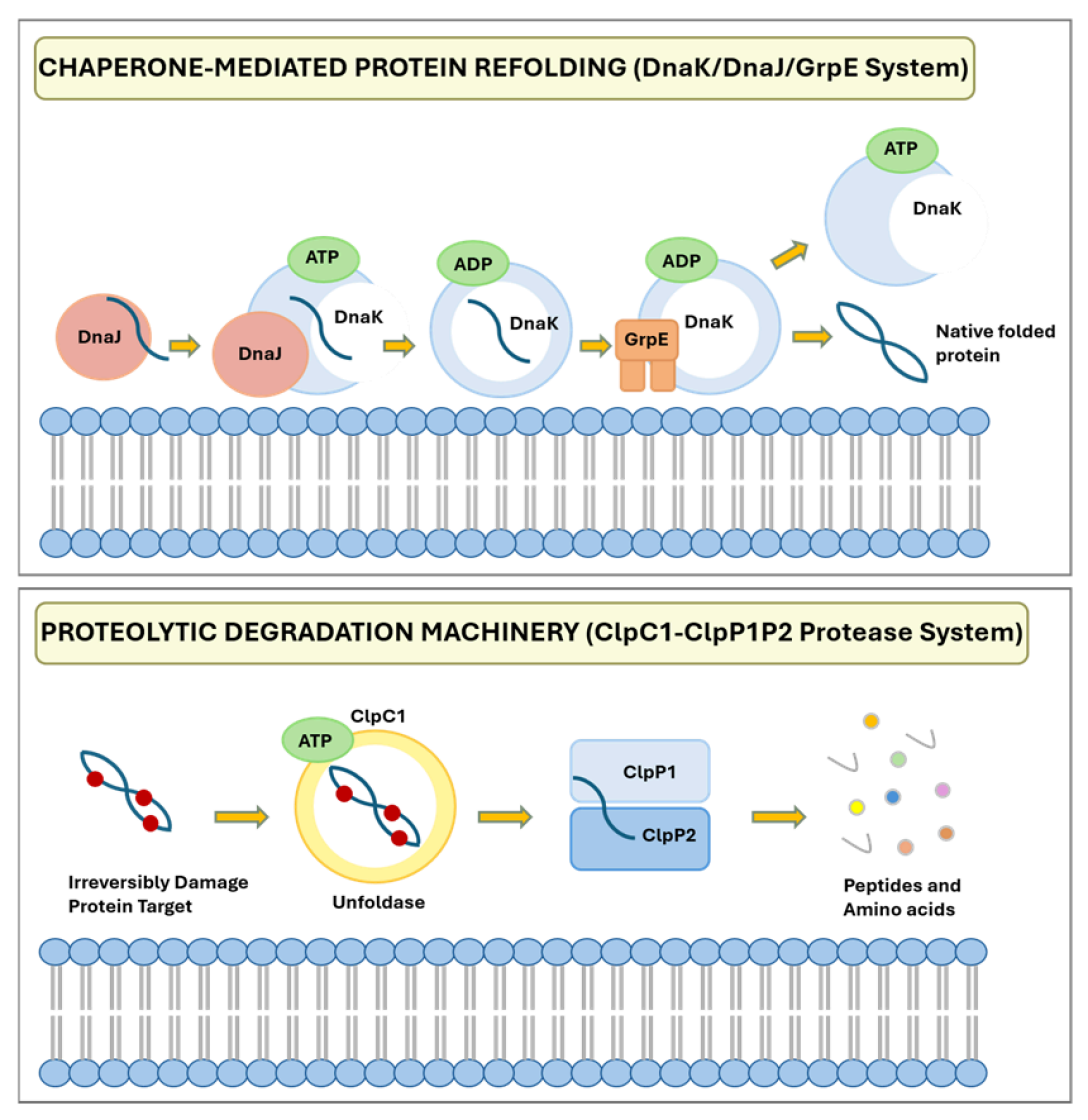
Major cytoplasmic protein quality control pathways: DnaK/DnaJ/GrpE system (chaperone-mediated refolding) and ClpC1-ClpP1P2 system (proteolytic degradation).

*M. tuberculosis* possesses several proteolytic systems, including Clp, FtsH, and eukaryotic-like proteases [67]. Among these, the ClpC1–ClpP1P2 complex is essential for bacterial growth and survival. ClpC1 recognizes and unfolds target proteins through ATP hydrolysis and translocates them to the proteolytic core ClpP1P2, where they are degraded into peptides. By maintaining protein homeostasis, this system represents an important drug target for anti-TB drug discovery [68] (Figure 2).

To date, there are no clinically approved antibiotics that directly act on the PQC system. However, several natural compounds have been identified that interfere with key components of this pathway, highlighting it as a promising target for antimicrobial drug discovery [69].

### 2.5. Alteration in Energy Metabolism

**Figure 3:**
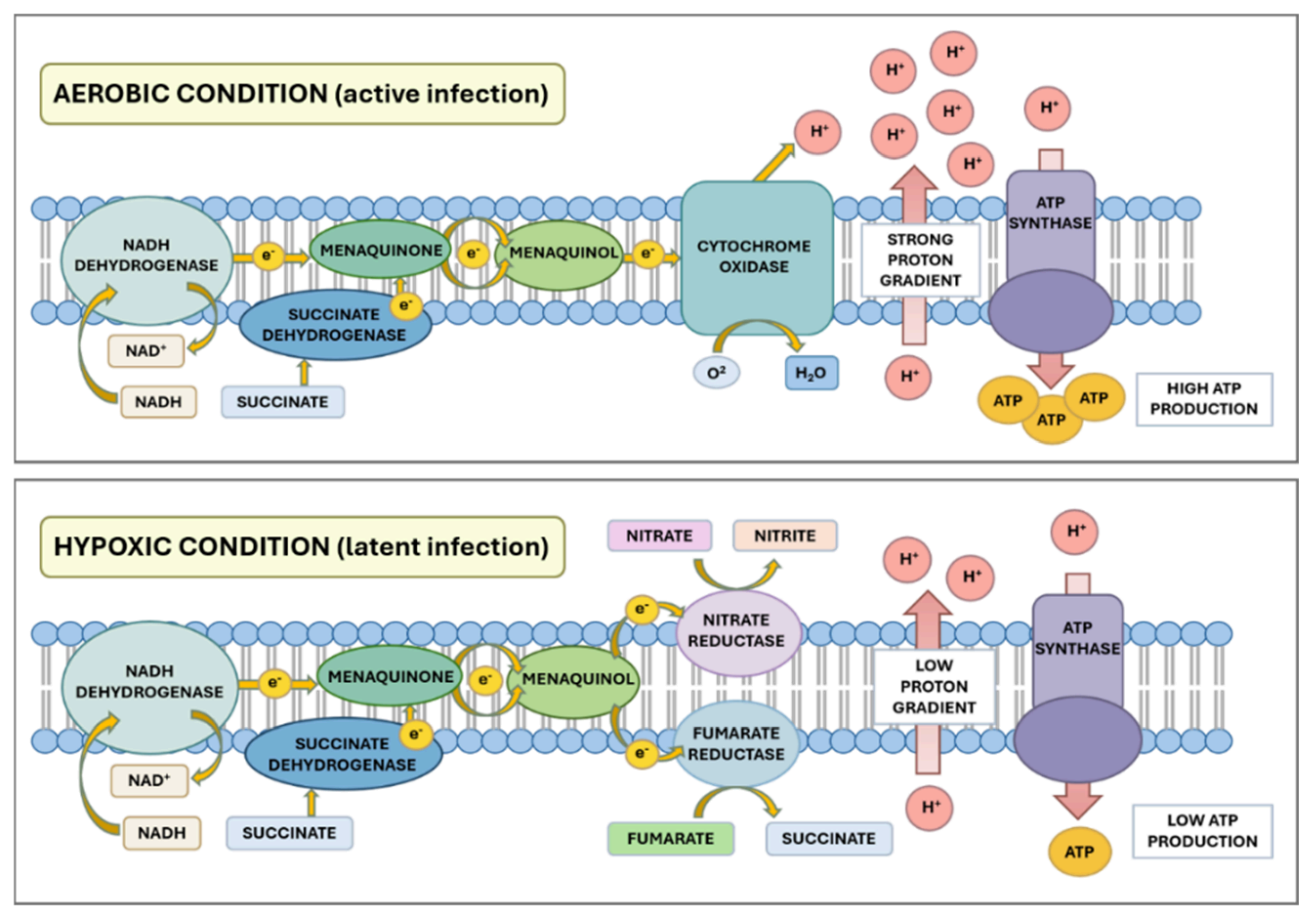
Metabolic adaptation and ATP production depending on oxygen availability. The switch of the electron transport chain from an aerobic state (active infection) to a hypoxic state (latent infection) is shown.

MTBC produces ATP through oxidative phosphorylation, a process that combines electron transport with ATP synthesis [70]. Electron transport in mycobacteria is initiated by NADH dehydrogenase (NDH) and succinate dehydrogenase (SDH), which transfer electrons from NADH and succinate to menaquinone (MK), forming menaquinol (MKH2). Subsequently, MKH2 delivers these electrons to various terminal complexes within the respiratory chain. Under aerobic conditions, cytochrome oxidase transfers electrons from MKH_2_ to oxygen, whereas under hypoxia, nitrate and fumarate reductases use nitrate and fumarate as alternative electron acceptors, producing nitrite and succinate, respectively. Electron transport generates a proton gradient across the membrane, which F_1_F_0_-ATP synthase uses to produce ATP [71]. This gradient and ATP production are maximal under aerobic conditions, but decrease during hypoxia, although a limited amount of ATP remains essential to maintain cell integrity and viability during latency [72] (Figure 3).

The electron transport chain has gained significance in the development of novel anti-TB compounds, such as bedaquiline and telacebec. These drugs act as inhibitors of ATP synthase and the mycobacterial cytochrome bc1 complex (QcrB subunit), respectively, inducing bacterial death [73,74].

## 3. Literature Search Strategy

A comprehensive literature search was conducted using PubMed, Scopus, and Web of Science databases. Articles published from January 2014 to present were included, using the following keywords: “microbial natural products,” “antimycobacterial activity,” “tuberculosis,” “*Mycobacterium tuberculosis*,” “microbial metabolites,” “antituberculosis agents,” “actinomycetes,” “fungi,” and “bioactive bacterial metabolites.” Additional studies were identified by reviewing the bibliographies of retrieved articles. The literature search identified approximately 243 potentially relevant articles. Titles and abstracts were reviewed for relevance, followed by a full-text assessment according to predefined inclusion and exclusion criteria. Studies reporting the antimycobacterial activity of purified natural products isolated from microorganisms against MTBC and providing experimental data supporting their activity were included. Studies investigating crude extracts, conference abstracts, or compounds evaluated exclusively against mycobacterial species outside of MTBC were excluded. Following this selection process, a total of 137 studies were included in the final review.

## 4. Antitubercular Activity of Microbial Natural Compounds

For several decades, natural products have been one of the most important sources for drug discovery, contributing significantly to the development of modern therapies. Indeed, a substantial portion of FDA-approved drugs are natural products or derived from naturally occurring structures. Classic examples include penicillin and streptomycin, secondary metabolites produced by the fungus Penicillium and the bacterium Streptomyces, respectively [75]. Among natural sources, products derived from microorganisms constitute one of the richest and most structurally diverse reservoirs of bioactive compounds. Bacteria and fungi can produce a wide range of secondary metabolites with antimicrobial, anti-inflammatory, immunomodulatory, and antioxidant properties, many of which have been successfully developed into clinically relevant drugs [76]. Microbial natural products have historically played a central role in the discovery of antibacterial agents and continue to provide valuable chemical frameworks for drug development. In recent years, interest in natural microbial compounds has further increased due to the growing prevalence of multidrug-resistant MTBC strains. Numerous lines of evidence have demonstrated the high antimycobacterial efficacy of several products derived from a wide variety of microorganisms, strengthening their relevance as a source of potential new anti-TB drugs [77]. The following sections provide a comprehensive overview of microbial natural products with antibacterial activity against human TB-causing mycobacteria, identified from 2014 to the present.

### 4.1. Actinomycetota-Derived Metabolites

Actinomycetes represent the most abundant microbial source of antimicrobial natural products. They constitute the largest phylum within the bacterial domain, in which the genus Streptomyces is the most numerous representative species. This genus includes more than 800 species, producing 80% of known bioactive compounds, including antibacterial molecules. The latter are described as stress metabolites under adverse environmental conditions, promoting competition with other microorganisms and favoring their survival [78]. In this context, a prime example of an actinomycete-derived antibiotic used in clinical practice for TB treatment is streptomycin. It is a secondary metabolite produced by *S. griseus* and was the first antibiotic to demonstrate activity against *M. tuberculosis*, thus establishing actinomycetes as a primary source for antimycobacterial drug discovery. This section reviews natural products obtained from actinomycetes, grouped according to their chemical structure into the following categories: (i) non-ribosomal peptides; (ii) polyketides; and (iii) other compounds.

#### 4.1.1. Actinomycete-Derived Non-Ribosomal Peptides

Non-ribosomal peptides are an important class of secondary metabolites produced primarily by bacteria and filamentous fungi [79]. These compounds are synthesized independently of the mRNA-ribosome system by large, multimodular enzyme complexes called non-ribosomal peptide synthetases [80]. Unlike ribosomal peptides, non-ribosomal peptides exhibit a high structural diversity, containing non-proteinogenic amino acids, D-amino acids, and numerous post-synthetic modifications, such as methylation, glycosylation, acylation, halogenation, and cyclization. This high structural complexity translates into a wide variety of biological activities, making these peptides a valuable source of bioactive molecules, including antimycobacterials [81].

Table 3 summarizes the non-ribosomal peptides derived from *Actinomycetota* sp., reporting for each compound the effective antimycobacterial concentration, the bacterial strains toward which the activity was evaluated and the related mechanisms of action, when available.

Pyridomycin is a cyclodepsipeptide produced by the genus Streptomyces. It is characterized by a 12-membered central ring composed of two pyridyl groups, a propionic acid and a 2-hydroxy-3-methylpent-2-enoic acid. This metabolite is known for its anti-TB potential, binding to the NADH cofactor-binding site of the InhA enzyme and thus blocking the biosynthesis of mycolic acids. In the study by Lee et al., approximately 4000 actinomycete strains were analyzed, identifying *Streptomyces* sp. strain W3009 as the producer of the well-known antimycobacterial agent pyridomycin. Through a metabolomic approach using molecular networking mass spectrometry, they identified seven novel pyridomycin derivatives. All compounds shared a common 3-hydroxypicolinic acid–L-threonine–3-(3-pyridyl)-L-alanine (3HP–T–3PA) core and differed in the structure of their 3-methylpentanoic acid side chain, conferring distinct linear and cyclic skeletons. Pyridomycin and its derivatives C, D, E, and F have a cyclic structure, while derivatives G, H, and I have a linear structure. To evaluate their antimicrobial potential, growth inhibition assays against *M. tuberculosis* H37Rv and inhibition of InhA activity were performed. Pyridomycin inhibited mycobacterial growth by 50% at a concentration of 1.08 µM and approximately 80% of InhA activity. The purified derivatives showed lower antibacterial potential. Besides pyridomycin, only derivatives E and F showed antibacterial potential with IC_50_ values of 6.90 and 9.14 µM, inhibiting InhA activity by approximately 70%. Despite their limited antibacterial activity (IC_50_ > 20 µM), derivatives C, D, G, H, and I showed InhA inhibitory activity ranging from 75 to 77%. This discrepancy could be attributed to the reduced permeability of the cell wall. The authors of this study will guide future studies aimed at improving the chemical properties of these derivatives to enhance their antibacterial activity [82].

Actinomycins are chromogenic cyclic peptides isolated from cultures of various *Streptomyces* species. They consist of a chromophore group and two pentapeptide chains, whose amino acid composition varies. To date, 30 natural and synthetic actinomycin analogues with anti-TB activity have been identified. Shah et al. purified actinomycin C1, actinomycin C2, and actinomycin C3 from cultures of *Streptomyces pratensis* (*S. pratensis*), collected from soil samples in the Kashmir region of the northwestern Himalayas. The fermentation broth of the isolated strain was subjected to ethyl acetate extraction and subsequently to various chromatographic techniques, resulting in the isolation of pure compounds. The antimycobacterial potential of actinomycin C1, C2 and C3 was evaluated against the replicating *M. tuberculosis* H37Rv strain, showing MIC values of 0.0625, 0.039 and 0.039 μg/mL [83], respectively. Subsequently, Rakhmawatie et al. investigated the antimycobacterial activity of another member of actinomycins, actinomycin D. In detail, this research group isolated 16 strains from the rhizosphere soil (mud) of a mangrove area on Pramuka Island, Indonesia. Forty-eight-hour broth cultures were extracted with ethyl acetate (1:1, *v*/*v*), and the dried crude extracts were tested against replicating *M. tuberculosis* H37Rv. Data showed that the supernatant extract derived from the culture of *Streptomyces parvus* (*S. parvus*) strain NBRC 14599 completely suppressed microbial growth at a dose of 25 μg/mL. Gas chromatography–mass spectrometry profiling of *S. parvus* NBRC 14599 supernatant extract yielded 16 fractions. Fractions F1 and F2 completely inhibited mycobacterial growth at 100 and 6.25 μg/mL, respectively. F2 yielded six additional peaks. The active peak completely inhibited mycobacterial growth at 0.78 μg/mL and was identified as actinomycin D by high-resolution mass spectrometry [84]. Finally, Qureshi et al. studied the antimycobacterial potential of two novel actinomycins, actinomycin-X2 (act-X2) and actinomycin-D (act-D), isolated from the *Streptomyces smyrnaeus* (*S. smyrnaeus*) strain UKAQ_23, collected from a mangrove sediment sample. Antimycobacterial activity was evaluated against replicating strains *M. tuberculosis* H37Ra, *M. bovis* BCG, and *M. tuberculosis* H37Rv. Data showed that act-X2 inhibited microbial growth with MIC values of 1.56, 1.56, and 2.64 µg/mL, respectively. At doses of 1.56, 1.56, and 1.80 µg/mL, act-D completely suppressed the growth of the aforementioned bacteria, respectively. *In silico* data showed that the protein kinase PknB was the primary target of both actinomycins. Binding energy calculations using generalized Born molecular mechanics/surface area demonstrated that act-X2 had a higher binding affinity for the enzymatic target. Therefore, act-X2 could represent a potential anti-TB drug [85]. Nurkanto et al. identified a compound structurally related to actinomycin D in *S. parvus* A612, exhibiting marked antimycobacterial activity. Genomic analysis identified an actinomycin D-related biosynthetic cluster (85% similarity), and the compound was structurally characterized by ^1^H NMR, ^13^C NMR, and ESI-MS spectra. *In vitro* assays on replicating *M. tuberculosis* H37Rv and *M. bovis* BCG were performed using a resazurin reduction assay, showing efficient growth inhibition with IC_50_ values of 0.07 and 0.02 μg/mL, respectively. To establish the mechanism of action, molecular docking studies were conducted between the actinomycin D-related compound and 21 *M. tuberculosis* targets. The results suggested a possible interaction with several targets, including InhA, CoaBC, shikimate kinase (MtSK), ASDH, BioA, and Ag85C. Experimental validation confirmed that the purified compound inhibits *M. tuberculosis* shikimate kinase (MtSK), with an IC_50_ of 41.2 μg/mL and a Ki of 58.0 μg/mL, showing superior activity compared to standard actinomycin D (IC_50_ = 89.9 μg/mL; Ki = 119.2 μg/mL). In contrast, no inhibitory activity was observed against the enzymes InhA, CoaBC, ASDH, BioA, and Ag85C. MtSK catalyzes the transfer of a phosphate group from ATP to shikimate, forming shikimate-3-phosphate, a key intermediate in the shikimate pathway. This metabolic pathway is essential for the biosynthesis of aromatic amino acids. Extensive molecular docking analysis revealed that the actinomycin D-related compound occupies the ATP-binding site of MtSK, forming predominantly hydrophobic interactions with residues involved in nucleotide recognition. The compound showed low cytotoxicity against human colorectal adenocarcinoma (DLD-I) cells, with a selectivity index maggiore di 1100 [86].

Cyclomarins are heptapeptide cyclopeptides that share a similar core structure but have crucial differences in their side chains, conferring different anti-TB activity. Ozeki et al. tested a collection of 10.080 actinomycete strains isolated from soil samples collected at various locations in Japan. The strains were grown in liquid medium for 48 h, and the supernatants were collected, extracted, and dried. These were analyzed for mycobacterial activity using the rBCG-MDP1-luc system, which identified 137 extracts with rBCG luciferase-suppressive activity up to a dilution of 1:1600. The antimycobacterial activity of all these extracts was further evaluated against the laboratory replicating strain H37Rv and two clinical strains of *M. tuberculosis* XDR. The results showed that only 41 of the tested extracts inhibited the microbial growth of the laboratory strain, and that at least one strain was resistant to dilutions of up to 1:200. Of the 41 extracts, 1904-1 was selected for its lower toxicity to eukaryotic cell lines and its strong antimycobacterial potential. The toxicity of compound 1904-1 was evaluated on human pulmonary adenocarcinoma A549 cells and mouse bone marrow-derived macrophages (BMDM) by 3-[4,5-dimethylthiazol-2-yl]-2,5 diphenyl tetrazolium bromide (MTT) assay. The extract showed no toxicity up to a concentration of 100 μg/mL. In terms of antimycobacterial activity, 1904-1 completely suppressed bacterial growth at concentrations of 0.13, 0.5, and 2.0–7.5 μg/mL against the rBCG luciferase system, laboratory strain H37Rv, and 2 *M. tuberculosis* XDR, respectively. The extract was characterized by electrospray ionization mass spectrometry and ^1^H and ^13^C NMR, identifying the active compound as cyclomarin A [87]. Cyclomarin A is a cyclopeptide secondary metabolite produced by *Streptomyces* sp. CNB-982 and first isolated by the Fenical and Clardy groups [88]. Its antimycobacterial activity, first investigated in 2008 [89], results from interaction with the essential ClpC1 ATPase, disrupting protein degradation and leading to mycobacterial cell death [90,91].

Echinomycin is a cyclic depsipeptide antibiotic that acts as a DNA intercalator, disrupting bacterial DNA replication and gene expression. Chen et al. evaluated the antimicrobial potential of echinomycin against replicating strains *M. bovis* BCG and *M. tuberculosis* H37Rv. The research team isolated the active compound from a culture of *Streptomyces fuscichromogenes* (*S. fuscichromogenes*) obtained from the Yaoli primeval forest in Jiangxi Province, China. The structure of echinomycin was determined by UV, 1H-NMR, 13C-NMR, and MS analysis. The compound completely suppressed the growth of *M. bovis* BCG and *M. tuberculosis* H37Rv at doses of 0.1 and 0.5 μg/mL, respectively, by acting as a DNA intercalator and impairing DNA replication and expression [92].

Rufomycins and ilamycins are cyclic heptapeptides characterized by an isoprenyl group attached to the nitrogen of the tryptophan ring. They are known to impair mycobacterial growth by interfering with ClpC1, a target essential for TB viability. Zhou et al. investigated the chemical and biological properties of rufomycins produced by *Streptomyces atratus* (*S. atratus*) MJM3502. Using column chromatography and semi-preparative HPLC, they isolated eight new rufomycin derivatives, whose structures were confirmed by ^1H NMR and high-resolution electrospray ionization mass spectrometry. Additional compounds, including rufomiazine, ilamycins B1 and B2, and a C-32 epimeric mixture of rufomycins I and II, were also isolated. Their antimycobacterial activity was evaluated against replicating *M. tuberculosis* H37Rv by MIC determination. The C-32 epimeric mixture of rufomycins I and II showed the strongest activity, with an MIC of 0.02 μM, followed by rufomycin NBZ8 at 0.03 μM, NBZ7 at 0.10 μM, NBZ5 at 0.11 μM, and ilamycin B2 at 0.20 μM. In contrast, NBZ4 and rufomiazine showed weak activity, with MIC values above 10 μM. Importantly, the rufomycins showed much weaker activity against non-replicating *M. tuberculosis* under hypoxic conditions, with MICs ranging from 4 to >10 μM. Binding studies by surface plasmon resonance confirmed interaction with ClpC1, with the most active compounds showing the strongest binding affinity. The compounds also displayed time-dependent bactericidal activity, with growth inhibition during the first seven days followed by mycobacterial cell death, and showed high selectivity toward mycobacteria in Vero cells [93]. Whereas Sun et al. isolated 12 novel ilamycin analogues from a culture of the marine actinomycete *S. atratus* SCSIO ZH16. The structures of the compounds were elucidated by high-resolution electrospray ionization mass spectrometry, 1D and 2D NMR, and single-crystal X-ray diffraction, confirming their identity. Their antimycobacterial potential against replicating *M. tuberculosis* H37Rv was detected by MIC determination. The ilamycins suppressed microbial growth at concentrations ranging from 0.0096 to 10 μM. In detail, ilamycin J showed the highest antimycobacterial activity, with an MIC of 0.0096 μM. Ilamycin L also showed marked activity with MIC values of 0.24 μM, while ilamycins I and R had MICs of 1.2 μM. Ilamycins K, P, and Q showed MIC values of 1.4 μM, followed by ilamycin M active at a concentration of 2.3 μM. In contrast, ilamycins G and H showed less efficacy, acting at a concentration of 9.5 μM, similarly to ilamycin N and ilamycin O, which completely inhibited bacterial growth at concentrations of 9.6 and 10 μM, respectively. Only the analogous ilamycin P showed significant cellular toxicity [94].

Atrovimycin is a cyclodepsipeptide isolated from species belonging to the genus Streptomyces. Structurally, atrovimycin has a locally hydroxylated acyl cinnamic acid chain, which is important for its biological activity. Liu et al. isolated atrovimycin from *Streptomyces atrovirens* (*S. strovirens*) strain LQ13 from soil in China. The compound completely suppressed the growth of *M. tuberculosis* H37Rv with an MIC of 1.88 μM (2.5 μg/mL). Its cytotoxicity was evaluated on human tumor and non-tumor cell lines. The compound did not exhibit significant toxicity on any of the cell lines tested. The researchers determined the biosynthetic pathway driving the rapid production of atrovimycin. They identified enzymes belonging to the cytochrome P450 family and epoxide hydrolases, responsible for introducing the vicinal dihydroxyl substitution. Furthermore, a P450 modifier enzyme responsible for the β-hydroxylation of the α-phenylalanine residue was characterized [95].

Ohmyungsamycins A and B, cyclic peptides isolated from the soil actinomycete strain SNJ042, were evaluated for their antimycobacterial activity against replicating *M. tuberculosis* H37Rv. Ohmyungsamycins A and B inhibited mycobacterial growth by 50% *in vitro* at doses of 0.06 and 0.12 μM, respectively. Furthermore, their antimycobacterial potential against the study strain was also evaluated in macrophages. Microbial load was significantly reduced when bone-marrow-derived macrophages (BMDM) infected with the mycobacterium were treated with ohmyungsamycins A and B at a dose of 10 μM. Subsequently, it was tested whether ohmyungsamycins A and B could enhance macrophage autophagy, thereby contributing to host antimicrobial defense. LC3 punctate formation and lipidation, indicators of autophagy induction, were assessed. The data showed that both compounds at a concentration of 10 μM significantly increased autophagy activation, comparable to the effects observed in response to rifampicin treatment. Using a *Drosophila melanogaster*-*Mycobacterium marinum* infection model, the compounds were shown to increase the survival of infected models, increase autophagy, and reduce microbial load. Activation of AMP-activated protein kinase (AMPK), required for phagosome maturation and macrophage inflammatory responses, was also demonstrated in response to treatment [96].

Atratumycin is a cyclic decapeptide consisting of 3-(2-methylphenyl)-2(E)-propenoic acid linked to an amino acid chain. Through genomic analysis of *S. atratus* SCSIO ZH16, isolated from the deep sea, Sun et al. identified and isolated atratumycin, a cyclic decapeptide. Its structure was revealed through several spectroscopic experiments, X-ray diffraction data, and the Marfey method. Atratumycin completely suppressed the growth of *M. tuberculosis* H37Ra and H37Rv at doses of 3.8 and 14.6 μM, respectively. Furthermore, it induced low cytotoxicity in eukaryotic cell models. Although its antimycobacterial efficacy is low, the therapeutic relevance of this compound is due to its very low cytotoxicity [97].

Ecumicin possesses a complex macrolactam structure composed of numerous amino acid residues, including N-methylated and non-proteinogenic amino acids, ester groups, and hydrophobic side chains. This structure confers high stability and enhanced interaction with cellular targets. Gao et al. extracted ecumicin from the culture medium of *Nonomuraea* sp. strain MJM5123, isolated from soil. Its antimycobacterial potential was evaluated against replicating and non-replicating strains *M. tuberculosis*, including H37Rv and related clinical isolates, while its cytotoxicity was evaluated against epithelial kidney monkey Vero cell lines and J774.1 murine macrophages. Ecumicin demonstrated bactericidal activity at concentrations of 0.34 and 1.5 μM, under replicative and non-replicative growth conditions, respectively. The compound exhibits low toxicity to eukaryotic cells, with a selectivity index greater than 640. Subcutaneous administration of ecumicin in a micellar formulation to murine models at a dose of 20–32 mg/kg of body weight improved the bioavailability of the compound, resulting in a reduction in lung bacterial load of 1.3 log_10_ at 20 mg/kg and 1.6 log_10_ at 32 mg/kg, respectively. Genomic analysis of laboratory-generated *M. tuberculosis* strains spontaneously resistant to ecumicin identified the ClpC1 ATPase complex as a potential target. PCR and sequencing data demonstrated the presence of mutations in the clpC1 (Rv3596c), ppsC (Rv2933), and espG3 (Rv0289) genes. The identification of ClpC1 as a target was further supported by drug-protein affinity assays. ClpC1, involved in protein degradation in association with the ClpP1P2 complex, was directly modulated by ecumicin. In the wild-type strain, ecumicin binds to ClpC1 and blocks normal protein degradation by uncoupling ATPase activity from proteolysis. In resistant strains, however, binding is reduced or absent, and the ClpC1-ClpP1P2 complex continues to function normally [98].

Taeanamide A is a cyclic macrolactone molecule composed of 10 amino acid residues, including Ser, Thr, Ala, Leu, Gly, Pro, and N-Ac-Dab, and a trans-unsaturated lipid chain. On the other hand, taeanamide B shares the same peptide sequence and lipid moiety but occurs in an acyclic form with a methyl-esterified terminal group. They were isolated from a culture of *Streptomyces* sp. strain AMD43, collected from a muddy soil sample from Anmyeondo, Korea. Taeanamide A showed low cytotoxicity against all tested cancer cell lines: human lung adenocarcinoma cells (A549), human colorectal carcinoma cells (HCT116), human breast cancer cells (MCF-7 and MDA-MB-231), human hepatocellular carcinoma cells (SK-Hep-1), and human gastric cancer cells (SNU638), with IC_50_ values > 20 μM, corresponding to a selectivity index > 0.74 against *M. tuberculosis* mc^2^6230. Taeanamide B showed higher cytotoxicity, with IC_50_ values ranging from 0.26 to 1.13 μM, corresponding to selectivity indices of 0.004-0.018. Both compounds exhibited moderate antimycobacterial activity against *M. tuberculosis* mc^2^6230, with IC_50_ values of 27 and 63 μM, respectively [99].

Thiolopyrrolone A and thiolutin belong to the class of dithiolopyrrolones. They consist of a bicyclic pyrrolinone-dithiol nucleus, with modifications affecting the N-4 and N-7 positions. Song et al. isolated two new thiolopyrrolone derivatives, thiolopyrrolone A and 2,2-dioxidothiolutin, and a known compound, thiolutin, from a culture of *Streptomyces* sp. strain BTBU20218885, isolated from a mud sample collected in the coastal region of Xiamen, China. Their chemical structures were determined by high-resolution electrospray ionization mass spectrometry and 1D and 2D NMR techniques. Thiopyrrolone A is a novel thiolutin analogue with a pseudo-trimeric structure comprising three thiolutin-like pyrrolone units, linked by disulfide and monosulfide bonds and bearing acetamide and N-methylamide groups. 2,2-Dioxidothiolutin is an oxidized thiolutin derivative containing two sulfoxide groups, while thiolutin is the parent compound with a simpler disulfide-containing structure. Among the isolated compounds, only thiolopyrrolone A and thiolutin showed antimycobacterial activity against replicating *M. bovis* and replicating *M. tuberculosis* H37Rv. Specifically, thiolopyrrolone A completely suppressed their growth at doses of 10 μM. On the other hand, thiolutin completely reduced the growth of these strains at concentrations of 0.3125 and 0.625 μM, respectively [100] (Table 3).

Among the compounds discussed, ecumycin represents a particularly promising candidate, combining potent bactericidal activity against both replicating and non-replicating *M. tuberculosis* (0.34 and 1.5 μM, respectively) with effective targeting of the essential proteostasis machinery ClpC1 and a high selectivity index (>640). Furthermore, subcutaneous administration at 20–32 mg/kg reduced lung bacterial burden by 1.3–1.6 log_10_ in mice, providing further support for its preclinical potential [98].

#### 4.1.2. Actinomycete-Derived Polyketides

Polyketides are a broad class of natural secondary metabolites characterized by high structural diversity and significant biological activity. They are organic compounds with oxidized carbon chains, often cyclic or partially cyclic, often including functional groups such as ketones, hydroxyls, conjugated double bonds, and macrolactam or macrolide systems. Polyketides are biosynthesized through the polyketide synthase pathway, multimodular enzymatic complexes that operate similarly to fatty acid synthesis. Synthesis occurs through the sequential condensation of activated acyl units, which are progressively assembled and modified along a growing chain.

Table 4 shows polyketides derived from actinomycetes, indicating the active dose at which the compound exhibits antimycobacterial activity, the strain against which the compounds are active, and any mechanisms of action. 

Niphimycins are macrolides composed of an alkylguanidyl side chain, known to alter the composition and integrity of cell membranes. Hu et al. collected *Streptomyces* sp. strain IMB7-145 from marine sediments. Through whole-genome sequence analysis, seven gene clusters responsible for the synthesis of type I polyketide synthase were identified. Detailed bioinformatic evaluations linked these genes to niphimycin biosynthesis. Guided by genomic analysis, four novel structural analogs of niphimycin, niphimycins C, D, E, and 17-O-methylniphimycin, were identified from large-scale cultures of *Streptomyces* sp. strain IMB7-145. The cytotoxicity of the compounds was evaluated on the human epithelial uterus tumor (HeLa) cell line, showing IC_50_ values ranging from 3.0 to 9.0 μM. Furthermore, they exhibit antimycobacterial activity against replicating *M. tuberculosis* clinical isolates, with MIC values ranging from 4 to 32 μg/mL [101].

Lincolnenins A–D are isomeric bianthracene compounds isolated from cultures of *Streptomyces lincolnensis* (*S. lincolnensis*). Mohamed et al. reported four new lincolnenins, A–D, whose structures were elucidated by spectroscopic analyses. The compounds exhibit atropisomerism arising from restricted rotation around the biaryl axis, with Lincolnenins A and B occurring as stable atropisomers, whereas C and D can interconvert. Among the four compounds, Lincolnenin A exhibited significant bactericidal activity against *M. tuberculosis* H37Ra, completely reducing the bacterial load at 0.9 μM [102].

Boromycin is a macrolide containing boron in a lipophilic macrocyclic lactone complex, which confers broad-spectrum antimicrobial activity. It is isolated from soil-derived cultures of *Streptomyces antibioticus* (*S. antibioticus*). Moreira et al. set out to demonstrate the antimycobacterial activity of boromycin against *M. tuberculosis* H37Rv and *M. bovis* BCG, highlighting its mechanism of action. First, the compound’s cytotoxicity was assessed on human hepatocellular carcinoma cells (HepG2 and Vero) as well as on human erythrocytes. Data showed moderate activity on the eukaryotic cell models tested, with CC_50_ values of 35 and 25 μM in HepG2 and Vero cells, respectively, and a HC_50_ of 40 μM in human erythrocytes. Boromycin exhibited potent antimycobacterial activity against replicating *M. tuberculosis* H37Rv and *M. bovis* BCG, with MIC_50_ values of approximately 80 nM and MIC_90_ and MBC_99_ values of 200 nM. Importantly, boromycin also exhibited bactericidal activity against nonreplicating bacilli under hypoxic conditions, with a 99% bactericidal concentration (WCC_99_) of 200 nM, indicating a replication-independent bactericidal action. The compound exhibited a selectivity index > 300. In response to boromycin, a rapid reduction in membrane potential was observed, accompanied by a subsequent decrease in intracellular ATP levels and cytoplasmic protein leakage, consistent with potassium ionophore activity, membrane depolarization, and altered cellular bioenergetics [103].

Steffimycins belong to the anthracycline class. They are considered atypical due to the presence of a ketone group at position C-10, two methoxy groups at positions C-2 and C-8, and a neutral deoxysugar linked to C-7, generally absent in conventional anthracyclines such as nogalamicin, aclacinomycin, and daunorubicin. In the study by Koyama et al., steffimycin, 10-dihydrosteffimycin, and 8-demethoxysteffimycin were evaluated against *M. bovis* BCG. They were isolated from high-yield cultures of *Streptomyces* sp. strain OPMA02852, collected from soil samples. The cytotoxicity of steffimycin, 10-dihydrosteffimycin, and 8-demethoxysteffimycin was evaluated on HeLa cells. All three compounds completely inhibited BCG viability at 1.4, 21.7, and 5.8 μM, respectively. Cytotoxicity against HeLa cells was lower, with IC_50_ values of 47.2, 68.4, and 57.0 μg/mL, respectively, corresponding to selectivity indices of 58.7, 5.5, and 18.1, respectively. These results indicate that steffimycin showed the highest selectivity, whereas reduction at C-10 substantially decreased selectivity. Modifications at C-8 and C-10 therefore significantly affect both antimycobacterial activity and selectivity [104].

Diazaquinomycins are classified as secondary metabolites, consisting of a 1,8-diazaanthraquinone nucleus with two additional nitrogen atoms that confer unique electron-transfer properties, contributing to their antibacterial activity. The presence of long lipophilic side chains in some structural variants enhanced antimicrobial potential. Mullowney et al. purified diazaquinomycins A, J, and H from the culture of *Micromonospora maritima* (*M. maritima*) strain B026, isolated from lake sediments. Their antimycobacterial potential was evaluated against *M. tuberculosis* H37Rv and related MDR clinical isolates and M. bovis. Using the MABA assay, diazaquinomycins H and J showed MIC values of 0.04 and 0.07 μg/mL against replicating *M. tuberculosis* H37Rv, respectively, while diazaquinomycin A showed an MIC of 0.10 μg/mL and maintained activity against MDR strains. Diazaquinomycin A was also evaluated against non-replicating *M. tuberculosis* using the low oxygen recovery (LORA) assay, showing an MIC of 0.72 μg/mL. However, it showed a MBC_99_ of 0.37 μg/mL under normoxic conditions, but no significant bactericidal activity under hypoxic conditions. Cytotoxicity tests showed that diazaquinomycins H, J, and A showed no cytotoxicity against the Vero cell line at the highest concentration tested, 28 μM. Regarding the mechanism of action, previous studies have suggested that diazaquinomycin A acts on the folate metabolic pathway by inhibiting thymidylate synthase (ThyA). However, Mullowney et al. found that diazaquinomycin A did not significantly inhibit ThyA of *M. tuberculosis* in biochemical assays. Furthermore, overexpression of ThyA in *M. tuberculosis* did not alter the compound’s MIC. Therefore, these results ruled out thymidylate synthase as the primary target and suggested that diazaquinomycin A acts through an alternative, yet unidentified, mechanism [105].

Murayaquinone and furaquinocin D are quinone-based metabolites. They are characterized by a quinone system consisting of an aromatic ring fused with conjugated carbonyl groups. This system interferes with cellular redox systems, promoting the formation of reactive oxygen species (ROS) and resulting in cellular oxidative stress. Bunbamrung et al. isolated these quinone-based compounds from the growth media of *Streptomyces* sp. strain TBRC7642, an endophytic actinomycete strain associated with the plant *Epipremnum aureum*. Both compounds showed antimycobacterial potential against *M. tuberculosis* H37Ra, although with different efficiencies. In fact, murayaquinone was the most active compound, completely inhibiting microbial growth at a concentration of 9.65 μM. On the other hand, the same effect was achieved with furaquinocin D at a dose of 129.40 μM. From the same microorganism, the research group also isolated iron-chelating compounds, such as methyl aeruginoate, (R)-desferri-ferritiocin methyl ester, and desferri-ferritiocin-4-hydroxyphenethyl ester. These completely suppressed mycobacterial growth at concentrations of 105.93, 139.51, and 34.91 μM, respectively. Among the isolated compounds, murayaquinone was the most active. Unfortunately, it exhibited significant cytotoxicity on eukaryotic models. However, the results suggest that murayaquinone may represent a promising structural scaffold for the development of new anti-TB agents [106]. Dinactin belongs to the family of polyether macrotetrolide ionophores, characterized by an oxygenated macrocyclic structure responsible for binding and transporting monovalent cations. Hussain et al. isolated dinactin from a culture of *Streptomyces puniceus* (*S. puniceus*), collected from soil samples collected in the Sonamarg Mountains, located in the northwestern Himalayas. Specifically, bioguided fractionation of the extract in ethyl acetate allowed for the isolation of the compound in question. Its structure was delineated by NMR and spectroscopic analyses, including High-Resolution Electrospray Ionization Mass Spectrometry and Fourier Transform Infrared Spectroscopy. Dynactin exhibited antimycobacterial activity against replicating *M. tuberculosis* H37Rv, with an MIC of 1 μg/mL determined by broth microdilution. Dynactin exhibited low cytotoxicity against the human embryonic kidney cell line HEK-293, with an IC_50_ of 80 μM, corresponding to a selectivity index of 80 [107].

Treponemycin is an 18-membered polyketide macrolide. Scientific evidence supports its antibacterial potential, as it inhibits the synthesis of threonyl-tRNA. Yassien et al. purified the macrolide by bioguided fractionation of the ethyl acetate extract of *Streptomyces mutabilis* (*S. mutabilis*) culture, collected from soil samples in Saudi Arabia. The structure of treponemycin was defined by comprehensive 1D and 2D NMR analysis and high-resolution electrospray ionization mass spectrometry. Treatment with treponemycin at a dose of 13.3 µg/mL completely reduced the *M. tuberculosis* load. The researchers observed that treponemycin production in the culture supernatant increased significantly by modifying the growth medium, specifically by using a starch-nitrate-based medium, replacing KNO_3_ with corn and yeast extract or tryptone, and removing CaCO_3_ and K_2_HPO_4_. Macrolide production was monitored by liquid chromatography–diode array detection–mass spectrometry [108].

Desertomycins belong to the class of polyether macrolides, characterized by a macrocyclic lactone ring enriched with numerous ether bonds and a high density of stereogenic centers. This structure can alter the functionality of biological membranes and cellular macromolecules. Using genomic analysis strategies, Wang et al. identified the biosynthetic gene clusters for desertomycin in the genome of *Streptomyces flavofungini* (*S. flavofungini*) strain TRM90047. After culturing the Streptomyces strain, the metabolites were purified by preparative HPLC, yielding desertomycin 44-1, desertomycin 44-2, and desertomycin A. Their structures were determined by 1D and 2D NMR spectroscopy. All purified metabolites exhibited antimycobacterial activity. Specifically, treatment with desertomycin 44-1, desertomycin 44-2, and desertomycin A at concentrations of 25, 50, and 25 µg/mL reduced microbial load by 50%. Molecular docking analyses revealed that isolated desertomycins interact with the proteins RPSL, RPLC, and CLPC1, with binding values ranging from −8.89 to −6.99 kcal/mol. RpsL and RplC are ribosomal proteins involved in protein synthesis, while ClpC1 is an ATP-dependent chaperonin that regulates protein homeostasis. These findings suggest that desertomycins may inhibit mycobacterial growth by acting on multiple essential cellular processes [109]. Braña et al. purified and characterized desertomycin G from the fermentation medium of *Streptomyces althioticus* (*S. althioticus*) MSM3, isolated from intertidal *Ulva* sp. collected in the Cantabrian Sea. The compound was obtained through C18 solid-phase extraction and bioassay-guided C18 HPLC fractionation, and its structure was elucidated by HRMS and 1D/2D NMR spectroscopy. Initially, the compound’s cytotoxicity was evaluated on A549 human lung cancer, DLD-1 colon cancer, and MCF-7 human breast adenocarcinoma cell lines, as well as against healthy breast fibroblasts. Data showed that desertomycin G reduced the viability of MCF-7 and A549 cell lines and healthy breast fibroblasts by 50% at concentrations of 6.7, 8.6, and 9.2 µM after 48 h, respectively. DLD-1 cells, however, were more resistant, showing IC_50_ values at doses greater than 10 µM. For its antimycobacterial counterpart, at an MIC of 16 µM, desertomycin G completely suppressed the growth of *M. tuberculosis* H37Rv and two related MDR strains [110].

Efomycin G, oxohygrolidin, and 29-O-methylabierixin were obtained from the actinomycete strain BCC72023, collected from a rice stalk (*Oriza sativa* L.) in Chumphon Province, Thailand. The crude extract of the bacteria-free culture was sequentially fractionated by chromatography on Sephadex LH-20, followed by semi-preparative and preparative reversed-phase HPLC, yielding the pure compounds. The structures of the compounds were subsequently determined by nuclear magnetic resonance spectroscopy, Fourier transform infrared spectroscopy, ultraviolet–visible spectroscopy, optical rotation measurements, and high-resolution electrospray ionization mass spectrometry. Efomycin G, oxoigrolidine, and 29-O-methylabierixin showed antimycobacterial activity against replicating *M. tuberculosis* H37Ra, with MIC values of 12.0, 50.0, and 50.0 μg/mL, respectively. Efomycin G exhibited cytotoxicity against human epidermoid carcinoma (KB) cells, human breast carcinoma (MCF-7) cells, human small cell lung carcinoma (NCI-H187) cells, and Vero cells, with IC_50_ values of 5.16, 7.86, 1.56, and 3.68 μg/mL, respectively, corresponding to a selectivity index of 0.31 based on the IC_50_/MIC ratio of Vero cells. Oxohygrolidine exhibited IC_50_ values of 4.70, 12.89, 2.96, and 15.47 μg/mL against the same cell lines, respectively, with a selectivity index of 0.31. 29-O-methylabierixin showed IC_50_ values of 16.86, 22.27, 10.12, and 4.29 μg/mL, respectively, corresponding to a selectivity index of 0.09 [111].

Aranciamycins are classified as anthracyclines. They have a planar aromatic tetracyclic nucleus of the anthraquinone type, containing quinone carbonyl groups responsible for their redox properties. Khalil et al. isolated Aranciamycin and its structural analogues I, J, and A from a culture of Australian marine *Streptomyces* sp. (CMB-M0150) by ethyl acetate extraction followed by chromatographic fractionation and subsequent purification by semi-preparative and preparative HPLC. All compounds exhibited moderate antimicrobial potential against *M. bovis*. In particular, aranciamycin I and J had an MIC of 10 μM, while aranciamycin and analogue A showed less activity with an MIC of 30 μM, highlighting a variability in potency related to the structural differences between the analogues. Furthermore, aranciamycin showed moderate cytotoxicity against a panel of human tumor cell lines, with IC_50_ values greater than 7.5 μM. Given their moderate antimycobacterial activity, aranciamycins may represent starting compounds for the development of new anti-TB drugs [112].

Gwanakosides are glycosylated polyketides characterized by an oxygen-rich polycyclic core derived from PKS, decorated with sugar moieties that contribute to their biological activity. Huynh et al. purified gwanakosides A and B from a co-culture of *Streptomyces* sp. strain GA02, isolated from mountain soil, and *Pandoraea* sp. strain GA02N. Spectroscopic analyses identified compound A as a dichlorinated naphthalene glycoside and compound B as a pentacyclic aromatic glycoside. NMR analyses established the chlorine positions and identified the sugar moiety as 6-deoxy-α-L-talopyranose, while the absolute configuration of compound B was proposed using DP4 calculations. Co-culture increased compound production approximately 100-fold. They showed cytotoxicity against various human tumor cell lines with IC_50_ ranging from 5.6 to 19.4 μM. Otherwise, only gwanakoside A at a concentration of 15 μg/mL reduced *M. tuberculosis* cell death by 50% [113].

Azalomycin B elaiophilin and 11,11′-O-dimethylelaiophilin are 16-membered macrodiolidic polyketides with a symmetrical, oxygen-rich lactone ring. The latter is an O-methylated analogue in which hydroxyl groups are replaced by methoxy groups. Both compounds were isolated from *Streptomyces* sp. BCC71188, a strain closely related to *Streptomyces samsunensis* (*S. samsunensis*) M1463T and *Streptomyces malaysiensis* (*S. malaysiensis*) NBRC 16446T was obtained from a soil sample in Nakhon Si Thammarat Province, Thailand. The crude extract of fermentation media exhibited activity against *M. tuberculosis* H37Ra, completely inhibiting its growth at a concentration of 6.25 μg/mL. Fractionation of the crude extract by Sephadex LH-20 column chromatography, combined with a bioassay-guided approach, led to the isolation of 19 compounds. Among them, two cyclic peptides, five macrolides, a novel naphthoquinone, C-nahuoic acid, several geldanamycin derivatives, cyclooctatin, germicidins A and C, actinoramide A, as well as abierixin and 29-O-methylabierixin were identified. The compounds were structurally characterized by ^1^H and ^13^C NMR and evaluated for cytotoxicity against NCI-H187, KB, MCF-7, and Vero cells, as well as for antimycobacterial activity against replicating *M. tuberculosis* H37Ra. The only compounds that showed effective biological activity were the macrolides azalomycin B and 11,11′-O-dimethylelaiophilin. They induced cytotoxicity with IC_50_ values lower than 1.82 μg/mL. Specifically, azalomycin B showed IC_50_ values of 0.46, 0.64, 1.82, and 0.62 μg/mL on the human small cell lung carcinoma NCI-H187, human oral cancer KB, human breast cancer MCF-7, and Vero cell lines, respectively. On the other hand, 11,11′-O-dimethylelaiophilin showed IC_50_ values of 0.65, 0.67, 1.43, and 1.13 μg/mL, indicating similar cytotoxic activity with slight variations between cell models. Both macrolides completely totally reduced the load of *M. tuberculosis* H37Ra at concentrations of 0.78 and 3.13 μg/mL, respectively [114].

Chrysomycin A is an angucycline characterized by four fused aromatic rings containing carbonyl and oxygenated groups. It was isolated from *Streptomyces* sp. OA161, obtained from soil in Thailand. The fermentation extract was purified by chromatography and HPLC, yielding two main fractions with retention times of 26.4 and 28.7 min. The fraction eluting at 28.7 min showed significant antimycobacterial activity against *M. tuberculosis* H37Ra and was subsequently purified and identified as chrysomycin A by NMR analysis. Its antimycobacterial activity was assessed against *M. tuberculosis* using the Resazurin Microtiter Assay (REMA), showing an MIC of 3.125 μg/mL. Time-course studies revealed a bactericidal effect, with no growth after 24 h of treatment at the MIC. The cytotoxicity of chrysomycin A was assessed on the rat skeletal muscle myoblast L6, human colorectal adenocarcinoma SW480, human embryonic kidney HEK-293, and human monocytic leukemia THP-1 cell lines at doses up to 10 times the MIC using MTT. The data show that the compound exhibits negligible toxicity in L6 and SW480 cells, with cell viability maintained even at the highest concentrations. Conversely, THP-1 cells showed greater sensitivity to the compound, with a more marked reduction in cell viability even at intermediate concentrations [115].

Frenolicins are aromatic naphthoquinone polyketides characterized by an oxidized 1,4-naphthoquinone nucleus, typical of redox-active systems and the main determinant of their biological properties. Frenolicins A and G were isolated from a culture of *Streptomyces* sp. strain TBRC17107 derived from a longkong bark-eating caterpillar. Their structures were determined by NMR and High-Resolution Electrospray Ionization Mass Spectrometry analysis. Both compounds induced mild cytotoxicity in human cell lines. Furthermore, both compounds exhibited antimycobacterial activity against *M. tuberculosis* H37Ra at a dose of 25.0 μg/mL [116].

Kimidinomycin is a complex polyketide, belonging to the 38-membered macrolide class, characterized by a side chain containing an N-methylguanidyl group and an oxygenated aromatic nucleus, which contributes to its chemical reactivity and potential redox properties. Hikima et al. purified this compound from the fermentation medium of the *Streptomyces* sp. KKTA-0263 strain. The fermentation medium was extracted by organic solvent extraction, followed by fractionation via HP20 column chromatography and subsequent preparative HPLC. This sequential approach allowed for the enrichment of the active fractions and the final purification of kimidinomycin. The latter inhibited the growth of *M. bovis* BCG at a concentration of 23.66 µg/mL [117].

Nybomycin belongs to the class of aromatic naphthoquinones. Its structure consists of a highly conjugated planar polycyclic core with quinone carbonyl groups that confer redox-active properties. Arai et al. purified nybomycin from a culture of marine-derived *Streptomyces* sp. strain MS44. The compound exhibited antimycobacterial activity against *M. bovis* BCG, completely suppressing growth of the mycobacterium at a dose of 1.0 μg/mL under both aerobic conditions of active growth and hypoxic conditions of dormancy. Evaluation of the mechanism of action indicated that the compound interferes with DNA structure, leading to cell death [118] (Table 4).

Overall, boromycin appears to be one of the most promising polyketides described, exhibiting potent antimycobacterial activity against both *M. tuberculosis* H37Rv and *M. bovis* BCG (0.2 μM) and a mechanism associated with disruption of membrane potential and depletion of intracellular ATP. However, its moderate cytotoxicity and lack of *in vivo* efficacy data highlight the need for further studies to evaluate its therapeutic potential. Nonetheless, boromycin represents a valuable scaffold for anti-TB drug development, providing analogues that retain its antimycobacterial activity and mechanism of action while enhancing cytotoxicity [103].

#### 4.1.3. Other Actinomycete-Derived Compounds

Among the various compounds isolated as secondary metabolites, structurally modified nucleosides are of particular interest. They are characterized by a nucleoside core derived from a nitrogenous base linked to a sugar, on which additional substituents, such as amino groups, modified sugars, and aromatic or acylated groups, are present, expanding their structural diversity and biological properties. Hosoda et al. isolated mavintramycins A, B, C, D, E, F, and G, and two other structurally related compounds, amycetin and plicacetin, from the culture broth of *Streptomyces* sp. strain OPMA4055, collected from marine sediments. The fermentation broth was extracted with ethyl acetate, and the resulting crude extract was subjected to a two-step chromatographic purification process to isolate the bioactive constituents. Structural characterization of the purified compounds was achieved by NMR analysis. All compounds shared a common backbone consisting of cytosine, amosamine, and amicetose units and differed in the chemical group attached at that position (R). Maventrimycins A–G, amycetin, and plicacetin were evaluated for antimycobacterial activity against replicating *M. bovis* BCG. Maventrimycin G and amycetin showed the highest activity, with MIC values of 0.39 μg/mL, followed by plicacetin with an MIC of 0.78 μg/mL. Maventrimycins A and F exhibited MIC values of 1.56 μg/mL, whereas maventrimycins B, C, D, and E showed lower activity, with MIC values of 6.25 μg/mL. Due to its high activity, maventrimycin A was further evaluated against *M. tuberculosis* H37Rv, showing an MIC of 0.38 μg/mL. Its cytotoxicity was assessed in both undifferentiated and differentiated THP-1 cells, with IC_50_ values > 10 μg/mL in both models, corresponding to a selectivity index greater than 26.3. To investigate its mechanism of action, macromolecular biosynthesis assays showed that maventrimycin A strongly inhibits protein synthesis. Resistance studies in *Mycobacterium avium* further supported this mechanism, as all resistant mutants harbored mutations in MAV101_007745, the gene encoding the 23S rRNA, with the 1992T>G mutation shared by all resistant strains. Furthermore, these mutants were also resistant to the structurally related compound amycetin [119].

(2S,2″S)-6-lavandulyl-7,4′-dimethoxy-5,2′-dihydroxyflavanone is a polyoxygenated flavanone featuring a C6–C3–C6 backbone, a lipophilic lavandulyl substituent at C-6, and hydroxyl/methoxyl groups on the aromatic rings, with (2S,2″S) stereochemistry. In the study conducted by Danh Cao et al., the compound was isolated from the culture of the strain *Streptomyces* sp. strain G248, obtained from the sponge *Halichondria panicea* (Pallas, 1766), collected on Son-Tra Island (Da Nang) in Vietnam. The fermentation broth was treated with XAD-16 resin, followed by fractionation using silica gel and Sephadex LH-20 chromatography. Active fractions were further purified by repeated chromatographic separations and preparative TLC. The compound’s structure was elucidated by HRESI-MS and NMR analyses. The antimycobacterial activity of the flavonone was evaluated against replicating *M. tuberculosis* H37Rv. At a dose of 48 μg/mL, the compound reduced the microbial load by 90%. Cytotoxicity data on KB, Hep-G2, human lung adenocarcinoma Lu-1 and MCF-7 showed IC_50_ values of 59.7, 32.0, 80.0 and 71.7 μg/mL, respectively [120].

2,4-Di-tert-butylphenol is an alkyl-substituted aromatic phenol (hindered phenol) with two tert-butyl groups at positions 2 and 4. The compound was isolated from the fermentation medium of *Streptomyces bacillaris* (*S. bacillaris*) strain ANS2, collected from soil samples. The culture was extracted using ethyl acetate. Using various chromatographic and spectroscopic analyses, 2,4-di-tert-butylphenol was identified and purified. The compound completely suppressed the growth of *M. tuberculosis* H37Rv and its MDR clinical isolate at concentrations of 48.47 and 242.37 μg/mL, respectively. Furthermore, 2,4-DTBP exhibited activity against the latent/non-replicating form of *M. tuberculosis* H37Rv in the Wayne hypoxic model, with an MIC of 100 μg/mL, indicating activity against both replicating and persister bacilli. Against the MDR isolate, 2,4-DTBP caused 78% and 74% inhibition at 100 and 50 μg/mL, respectively. Molecular docking analyses indicated that the compound positions itself in the enzymatic pocket of lysine aminotransferase (LAT), with a predicted binding energy of −5.3 kcal/mol, suggesting interference with amino acid metabolism and supporting LAT as a potential molecular target [121].

Lassomycin belongs to the class of ribosomally synthesized and post-translationally modified peptides and to the subclass of lassopeptides. It consists of a cyclic ring with a terminal peptide chain spanning it. Gavrish et al. purified the compound from the culture of the strain *Lentzea kentuckyensis* (*L. kentuckyensis*), collected from soil samples. Its antimycobacterial potential was evaluated on a wide variety of *M. tuberculosis* strains, both standard and clinical isolates. Specifically, against the reference strain of *M. tuberculosis* H37Rv, it showed MIC values ranging from 0.78 to 1.56 μg/mL. Similar activity was recorded for strains resistant to anti-TB drugs such as isoniazid, rifampicin, streptomycin, ethambutol, pyrazinamide, and fluoroquinolones. The MIC values obtained ranged from 0.78 to 3.1 μg/mL, indicating that resistance mechanisms to conventional anti-TB drugs did not compromise the functionality of lassomycin. Furthermore, the compound showed greater activity against *M. tuberculosis* mc^2^6020 strains, exhibiting MIC values ranging from 0.39 to 0.78 μg/mL. To investigate the mechanism of action of lassomycin, resistant mutants of *M. tuberculosis* were generated by prolonged exposure of the bacterium to the compound and subsequent selection of colonies growing on lassomycin-containing medium. Genome sequencing of the resistant mutants revealed that all had mutations in the clpC1 gene, which encodes the ClpC1 protein. This result was confirmed by biochemical tests. The findings demonstrated that lassomycin increased ATP hydrolysis 7–10-fold at a concentration comparable to the MIC, suggesting that dysregulation of its ATPase activity underlies the bactericidal effect [122].

Quigley et al. screened fermentation extracts from 10,241 bacterial strains for selective activity against *M. tuberculosis*. Bioactive extracts were fractionated by HPLC, leading to the isolation of four compounds whose structures were elucidated by 1D- and 2D-NMR: the novel peptide siderophores amycobactin, kitamycobactin, and streptomycobactin, and the previously characterized marfomycin D. Amycobactin possesses a cyclic macrolactone structure and was produced by Amycolatopsis, kitamycobactin has a looped peptide structure and was isolated from Kitasatospora, while streptomycobactin is a linear 20-amino-acid peptide obtained from Streptomyces. All three producers belong to the phylum Actinomycetota, whereas marfomycin D is a previously known non-ribosomal peptide. Limited cytotoxicity was recorded for the HepG2 and mouse embryonic fibroblast NIH/3T3 cell lines. Specifically, kitamycobactin showed lower toxicity with TC_50_ values ≥ 100 µg/mL, while amycobactin, streptomycobactin, and marfomycin D showed TC_50_ values in the range of 16–32 µg/mL. On the other hand, the compounds exhibited potent antimycobacterial activity. Streptomycobactin and kitamycobactin completely reduced the load of *M. tuberculosis* mc^2^6020 and H37Rv at doses of 0.03 and 0.06 µg/mL. Amycobactin showed more moderate activity with MIC ranges of 4–8 µg/mL, while marfomycin D showed variable activity between the two strains, recording MICs of 1 µg/mL for the mc^2^6020 strain and 0.03 µg/mL for the H37Rv strain. Killing kinetic studies showed that kitamycobactin showed mainly bacteriostatic activity during active growth, but moderate bactericidal activity in the stationary phase. Streptomycobactin and kitamycobactin showed bactericidal activity in the exponential phase, while only kitamycobactin maintained bactericidal activity even in the stationary phase. Marfomycin D showed a transient bacteriostatic effect during the exponential phase, with loss of efficacy after seven days. To identify the compounds’ mechanism of action, the idea was to select resistant mutants of *M. tuberculosis* through continuous exposure to the various compounds. For streptomycobactin, the generation of mutants was unsuccessful; so, its mechanism of action was not elucidated. Given the structural similarity of kitamycobactin and lassomycin, mutants of the latter were used to assess the presence of cross-resistance. Cross-resistance was present, therefore, kitamycobactin acts on the ClpP1P2C1 system. For amycobactin, the selection of *M. tuberculosis* mutants was also unsuccessful. Therefore, the researchers exploited *M. smegmatis*, obtaining two resistant mutants characterized by deletions in the secY gene. The production of these mutations in the *M. tuberculosis* strain confirmed the role of SecY, a component of the Sec secretion system, as a target of amycobactin [123] (Table 5).

In conclusion, kitamycobactin emerges as a very promising antimycobacterial compound. Potent activity against *M. tuberculosis* H37Rv and mc^2^6020 (0.06 and 0.03 μg/mL, respectively) was demonstrated, with bactericidal activity during both exponential and stationary growth phases by disrupting the ClpP1P2C1 proteolytic system. Furthermore, its relatively low cytotoxicity (TC_50_ ≥ 100 μg/mL) further supports its therapeutic potential. However, no *in vivo* efficacy data are available, and further studies are therefore needed to validate its antimycobacterial activity and evaluate its therapeutic potential *in vivo* [123].

### 4.2. Other Bacterial Metabolites

In addition to actinomycete-derived compounds, several non-actinomycete bacteria produce secondary metabolites with significant activity against MTBC. Although less studied, these organisms nevertheless represent an emerging reservoir of structurally diverse antimicrobial agents with a variety of mechanisms of action. Ling and colleagues used an innovative device, called the iChip, which allows them to cultivate soil microorganisms that are normally uncultivable in the laboratory. Extracts from 10,000 isolates obtained by growing on the iChip were evaluated for their antibacterial activity. Among these, the extract derived from *Eleftheria terrae* (*E. terrae*) showed effective activity. Teixobactin was isolated from the active fraction, and its structure was determined by mass spectrometry and NMR studies. Teixobactin exhibited potent activity against replicating *M. tuberculosis*, with a MIC below 1 μg/mL. No teixobactin-resistant *M. tuberculosis* mutants could be obtained, even when bacteria were exposed to 4× the MIC. Its activity is attributed to inhibition of cell-envelope biosynthesis through binding to conserved pyrophosphate-containing lipid intermediates. In *M. tuberculosis*, teixobactin is proposed to target decaprenyl-coupled precursors involved in peptidoglycan and arabinogalactan biosynthesis, providing a mechanistic explanation for its antimycobacterial activity [124].

Imai et al. evaluated the antimycobacterial efficacy of evybactin and its mechanism of action against strains of *M. tuberculosis*. The compound was isolated from the culture of *Photorhabdus noenieputensis* (*P. noenieputensis*) strain DSM 25462, a bacterial symbiont of entomopathogenic nematodes. The bacterium was cultured for eight days, after which the fermentation broth was extracted using XAD16N resin and purified by reversed-phase HPLC, yielding evybactin at 92% purity. Its structure was elucidated by HR-LC-MS and multidimensional NMR, revealing a cyclic depsipeptide containing 11 amino acid residues, including N-methylhistidine, N-formyltryptophan, and two β-aspartates. The absolute configurations of the amino acids were determined by Marfey’s derivatization method. Evybactin showed no toxicity against human HepG2, FaDu, and HEK293 cells at concentrations below 128 μg/mL. Furthermore, it completely suppressed the growth of replicating *M. tuberculosis* strains H37Rv and mc26020 at a concentration of 0.25 μg/mL. To determine the mechanism of action, *M. tuberculosis* H37Rv strains were selected and subsequently subjected to genomic analysis. Specifically, the strains were exposed to high concentrations of evybactin, 10 times the MIC, to select spontaneously resistant mutants. Genomic sequencing of the mutants revealed mutations in the BacA gene, which encodes a transporter involved in the compound’s entry into cells. This transporter is not an essential enzyme for bacterial life; in fact, loss of the transporter would not necessarily cause bacterial death. To identify the compound’s true target, TB strains were continuously treated at higher doses, 100 times the MIC, to avoid mutations in the bacA gene. Sequencing data showed point mutations in the gyrA gene, which encodes the A subunit of DNA gyrase. The target was confirmed by biochemical assays. The crystallographic structure of the DNA gyrase and evybactin complex showed that the compound occupies a site on DNA gyrase distinct from that occupied by fluoroquinolones and binds to the winged-helix domain of the GyrA subunit. This location highlights the novel mechanism of action and the lack of cross-resistance with fluoroquinolones [125] (Table 6).

Among the mentioned products, evybactin represents the most promising antimycobacterial compound. It exhibits potent activity against *M. tuberculosis* H37Rv and mc^2^6020 (0.25 μg/mL), by binding to the GyrA subunit of DNA gyrase at a site distinct from that of fluoroquinolones, suggesting a reduced potential for cross-resistance. It showed a favorable cytotoxicity profile against several eukaryotic cell lines at doses lower than 128 μg/mL [125]. However, the lack of *in vivo* efficacy data highlights the need for further studies to validate its therapeutic potential and pharmacological properties.

Although actinomycetes represent the largest and most studied source of natural metabolites with antimycobacterial activity, recent studies have highlighted the emerging role of non-actinomycete bacteria as alternative reservoirs of novel bioactive molecules. Even if historically less explored, these microorganisms have demonstrated high chemical diversity and mechanisms of action, including the inhibition of essential cell-wall biosynthetic pathways and the selective targeting of key intracellular enzymes, such as DNA gyrase. The structural diversity of these metabolites and their ability to act on vital bacterial targets underscore the potential of unconventional bacterial sources in the discovery of novel therapeutic agents against TB.

### 4.3. Fungal-Derived Metabolites

Fungi represent another important source of bioactive secondary metabolites. These metabolites are not directly involved in fungal life processes but are important for ecological interactions through mechanisms of microbial competition, defense, and the establishment of symbiotic relationships [126]. Due to their structural diversity and broad range of biological activities, these compounds have been widely exploited in various fields, including medicine, agriculture, and industry [127]. Among the most representative examples are penicillins and cephalosporins, antibiotics currently used clinically against a broad spectrum of bacteria [128].

Luo et al. isolated 12 compounds from the marine fungus *Aspergillus* sp. SCSIO Ind09F01, including diketopiperazine alkaloids, fumiquinazoline alkaloids, and tetracyclic triterpenoids. Their structures were elucidated by NMR and ESI-MS and comparison with known compounds. Among the isolates, gliotoxin, 12,13-dihydroxyfumitremorgine C, and helvolic acid showed significant biological activity. Gliotoxin is an epipolythiodioxopiperazine (ETP) containing an intracyclic disulfide bridge, 12,13-dihydroxyfumitremorgine C is a prenylated indole alkaloid, and helvolic acid is a fusidan-type steroidal triterpenoid. Gliotoxin, 12,13-dihydroxy-fumitremorgine C, and helvolic acid inhibited the growth of *M. tuberculosis* H37Rv with MIC_50_ values of <0.03, 2.41, and 0.894 μM, respectively. Among the purified products, only gliotoxin induced significant cytotoxicity against the human chronic myelogenous leukemia cell line K562, the human lung adenocarcinoma cell line A549, and the human hepatocellular carcinoma cell line Huh-7, showing IC_50_ values of 0.191, 0.015, and 95.4 μM, respectively [129].

Andrioli et al. evaluated six fungal metabolites isolated from three different fungal species: austdiol, austdiol diacetate, mycoleptone A, and eugenitin from endophyte *Mycoleptodiscus indicus* (*M. indicus*), emodin from *Penicillium citrinum* (*P. citrinum*), and a δ-lactam derivative from *Humicola grisea* (*H. grisea*). Metabolites were extracted from the fermentation medium with ethyl acetate. The obtained crude extract was purified by chromatography on Sephadex LH-20 and silica, leading to the isolation of the above compounds, which were characterized by NMR and mass spectrometry. Austdiol, austdiol diacetate, and mycoleptone A are oxygenated fungal polyketides, whereas eugenitin and emodin are quinone derivatives. Cytotoxic activity was assessed on mouse macrophages RAW 264.7 using the MTT assay. All products exhibited CC_50_ values greater than 100 μM. At the highest dose, only austdiol, mycoleptone A, and emodin induced weak cellular toxicity of less than 30%. Antimycobacterial potential was assessed against replicating *M. bovis* BCG and *M. tuberculosis* strains H37Rv, M442, and M299. Findings showed variable antimycobacterial potential. In detail, emodin showed the greatest antimycobacterial potency, with a MIC_50_ of 0.4 µM against *M. bovis* BCG and values between 5.0 and 8.2 µM in strains of *M. tuberculosis*. Mycoleptone A showed good activity against *M. bovis* BCG with a MIC_50_ of 1.7 µM, while it exhibited MIC_50_ values between 9.2 and 37.4 µM among the different TB strains. Eugenitin did not significantly impact the growth of *M. bovis* BCG, while for the TB strains H37Rv, M442, and M299, the MIC_50_ values were 79.1, 81.2, and >100 µM, respectively. The δ-lactam derivative showed limited activity, exhibiting MIC_50_ of 20.5 µM for *M. bovis* BCG, and >100 µM for all TB strains. Conversely, austdiol exhibited the opposite pattern, showing MIC_50_ of 11.7 µM for the BCG strain, and between 10.8 and 33.4 µM for the TB strains. Its diacetate variant showed reduced activity. Specifically, it showed MIC_50_ of 23.4 µM for *M. bovis* BCG; otherwise, MIC_50_ of 93.4 and >100 µM were recorded for strains M299, H37Rv, and M442, respectively [130].

Calixto et al. purified the macrolide (R)-(+)-lasiodiplodin from the culture of the endophytic fungus *Sordaria tamaensis* (*S. tamaensis*), obtained from the leaves and stem of the *Tocoyena bullata* plant. The fungal fermentation medium was extracted with ethanol and fractionated into hexane, ethyl acetate, butanol, and aqueous fractions. The ethyl acetate fraction (Tb1A) showed the highest antimycobacterial activity, with the active compound identified by a major chromatographic peak at 17.21 min. MS and NMR analyses identified the compound as (R)-(+)-lasiodiplodin, a 12-membered macrolide containing a resorcinol moiety and a chiral center with the R configuration. Its cytotoxic activity on RAW 264.7 macrophages and antimycobacterial activity against strains *M. bovis* BCG and *M. tuberculosis* H37Rv and M299 were evaluated. The pure compound induced 50% cell viability at a dose of 242.6 µg/mL. A significant antimycobacterial activity was found. The macrolide (R)-(+)-lasiodiplodin showed MIC_50_ values of 6.7, 60.4, and 92.2 µg/mL on BCG strain and *M. tuberculosis* H37Rv and M299, respectively. In C57BL/6 mice infected with the hypervirulent M299 strain, intraperitoneal treatment with a dose of 20 mg/kg for 15 days led to a reduction in bacterial load of 1.5 log_10_ CFU. Furthermore, a 20% reduction in the area affected by pneumonia, a strong decrease in relative lung weight, a drastic reduction in the recruitment of neutrophils, inflammatory monocytes, and dendritic cells, and, finally, a blockade of the production of pro-inflammatory cytokines (IL-6, TNF-alpha, and IL-17A) were observed. Chemogenomic and molecular docking studies have identified class II fructose-1,6-bisphosphate aldolase (FBPA) as a potential macrolide target in *M. tuberculosis*. FBPA is an enzyme involved in glycolysis and catalyzes the reversible conversion of dihydroxyacetone phosphate and glyceraldehyde-3-phosphate to fructose-1,6-bisphosphate. Both conformations bind in the active site through hydrogen bonds with residues such as Gly254, Ser255, and Asn274, and hydrophobic/van der Waals interactions with residues such as Gln51, His252, and Val275. Furthermore, the methoxy group present in the (R)-(+)-lasiodiplodin structure interacts with the Zn^2+^ ion in the catalytic site of FBPA, blocking the enzyme’s activity [131].

In the study conducted by Isaka et al., secondary metabolites produced by *Ganoderma orbiforme* (*G. orbiforme*), isolated from a dead oil palm trunk on a plantation in Klong Thom, Thailand, were isolated and characterized. The fungal strain was grown on a large scale, and the resulting fermentation medium was extracted with methanol or acetone. The resulting crude extracts were fractionated using solvents of different polarities, such as hexane and ethyl acetate. The organic fractions were concentrated and further purified by silica column chromatography and preparative reversed-phase HPLC. Among the numerous isolated compounds, several different bioactive lanostanoids were detected. They belong to the class of lanostane triterpenes. These compounds are characterized by a C30 tetracyclic skeleton of the lanosta-8,24-dien-26-oic type, with a side chain at C-17 and a carboxyl group at C-26. The structural differences between the various analogues are mainly due to the presence of hydroxyl, acetoxy, methoxy, and carbonyl groups at the C-3, C-7, C-15, and C-22 positions. The cytotoxic and antimycobacterial activities of these compounds were evaluated on the Vero cell line and on the replicating *M. tuberculosis* H37Ra strain. The compounds that showed the best selectivity index were (24E)-7α-Hydroxy-3-oxolanosta-8,24-dien-26-oic acid, (24E)-3β,15α-Diacetoxylanosta-7,9(11),24-trien-26-oic acid and ganorbiformin C. The first had an IC_50_ value of 103 µM in eukaryotic cells, compared to an MIC of 3.13 µg/mL against the *M. tuberculosis* H37Ra strain. (24E)-3β,15α-Diacetoxylanosta-7,9(11),24-trien-26-oic acid induced a 50% reduction in cell viability at a dose of 32 µM and suppressed mycobacterial growth at a concentration of 0.391 µg/mL. Finally, ganorbiformin C impacted the viability of Vero cells with an IC_50_ of 87 µM while showing a MIC of 1.56 µg/mL against the H37Ra strain [132].

The same experimental design was used by the same research group to purify and analyze the metabolites of the culture of the fungal strain *Ganoderma* sp. strain BCC 16642, obtained from a wood sample collected in Doi Suthep-Pui National Park in Thailand. Again, the fungal culture was fermented on a large scale, extracted with organic solvents, and the resulting metabolites were purified by silica chromatography and preparative reversed-phase HPLC. Forty-two metabolites were purified and classified as lanostane triterpenes. The novel compounds with a better selectivity index were (24E)-3β,15α-diacetoxy-7α-hydroxylanosta-8,24-dien-26-oic acid and 3β,15α-diacetoxylanosta-8,24-dien-26-oic acid. Their cytotoxic potency was evaluated on the Vero cell line, while the antimycobacterial activity against replicating *M. tuberculosis* H37Ra. (24E)-3β,15α-diacetoxy-7α-hydroxylanosta-8,24-dien-26-oic acid reduced cell viability by 50% at a dose of 32 μM and totally inhibited microbial growth at a MIC of 1.56 μg/mL. In contrast, 3β,15α-diacetoxy lanosta-8,24-dien-26-oic acid impacted the viability of Vero cells and the bacterial strain with IC_50_ values of 22 μM and MIC of 0.781 μg/mL [133] (Table 7).

Overall, (R)-(+)-lasiodiplodin emerges as the most promising fungal metabolite described, combining significant *in vitro* antimycobacterial activity (MIC_50_ = 6.7 μg/mL against *M. bovis* BCG and 60.4 μg/mL against *M. tuberculosis* H37Rv), limited cytotoxicity (CC_50_ = 242.6 μg/mL), and *in vivo* efficacy, with a 1.5 log_10_ reduction in pulmonary bacterial load. Although FBPA has been proposed as a potential target, further studies are required to confirm its mechanism of action. Nevertheless, (R)-(+)-lasiodiplodin represents a promising scaffold for developing analogues with improved cytotoxicity and selectivity [131].

## 5. Conclusions

**Figure 4:**
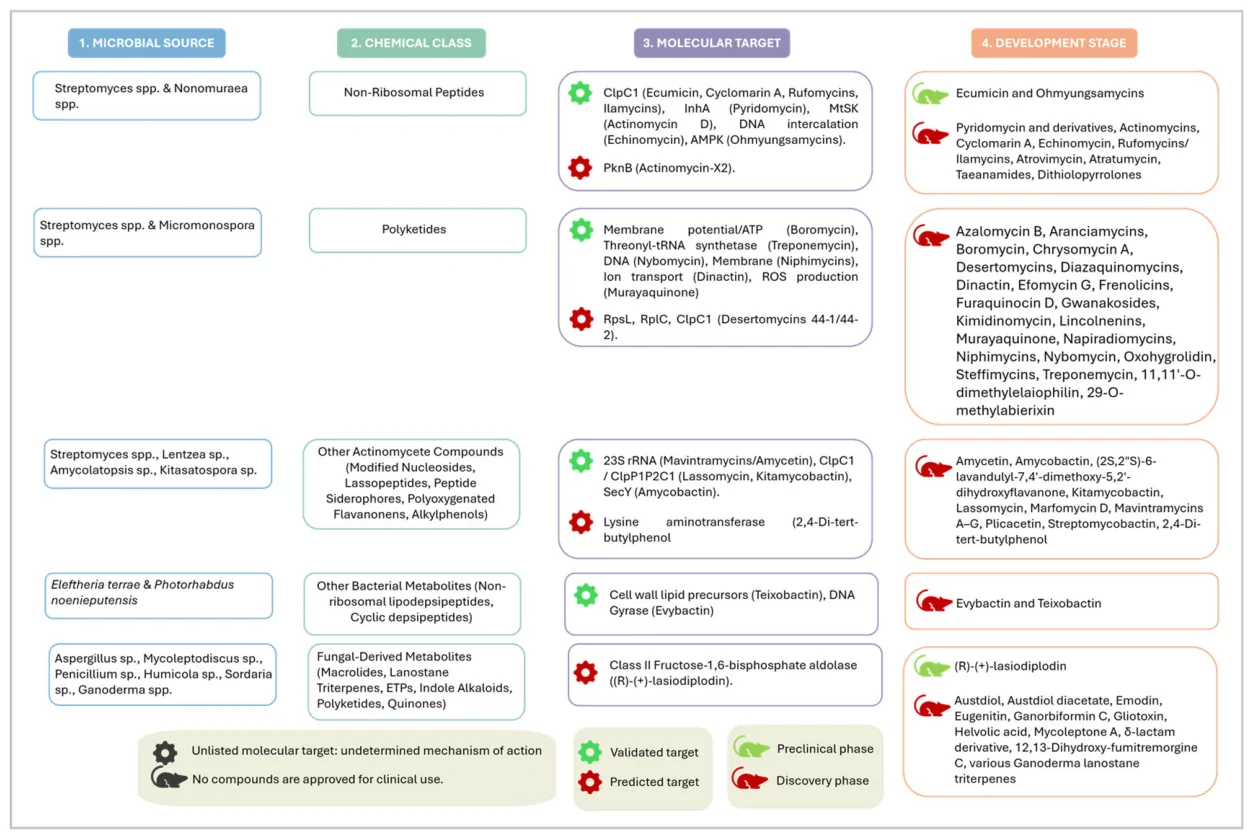
Schematic overview summarizing the major classes of microbial natural products active against MTBC, categorized by microbial source, chemical class, molecular target, and current stage of development.

The rapid emergence of MDR and XDR TB strains has rendered current therapeutic regimens increasingly ineffective, underscoring the urgent need for innovative drugs with alternative mechanisms of action. In this context, microorganisms, including bacteria and fungi, represent an important source of natural products that exhibit potent antimycobacterial activity combined with low cytotoxicity. Several promising secondary metabolites were highlighted in this review (Figure 4). Among the most notable examples is ecumicin, isolated from the fermentation medium of *Nonomuraea* sp. This compound exhibited potent bactericidal activity under both replicating and non-replicating conditions, with a selectivity index greater than 640, and a novel mechanism of action, with the ClpC1 ATPase complex identified as its molecular target. Potent antimycobacterial activity, low cytotoxicity, and target specificity represent key characteristics for the development of effective therapies against drug-resistant TB.

The natural products discussed in this review represent promising compounds that merit further *in vivo* evaluation to characterize their pharmacokinetic properties, bioavailability, efficacy, and safety. However, despite the promising antimycobacterial activity of several microbial natural products, their translation into clinically useful anti-TB agents remains challenging [134]. Major limitations include unfavorable pharmacokinetic profiles, such as poor solubility, chemical instability, limited bioavailability, and suboptimal tissue penetration, compromising *in vivo* efficacy despite demonstrated potent *in vitro* activity. Another critical issue concerns safety, as potent antimycobacterial activity does not necessarily guarantee an adequate therapeutic window. Systematic evaluations of cytotoxicity, off-target effects, metabolic stability, and *in vivo* tolerability will therefore be necessary. The limited availability of some microbial metabolites also represents a major obstacle to preclinical development. Low fermentation yields, difficulties in isolation and purification, and the complexity of large-scale production can limit the availability of material for pharmacological studies and subsequent clinical development. In this context, optimization of fermentation processes, metabolic engineering, heterologous expression, and genome-mining strategies could help improve the production and availability of these compounds.

Finally, clinical translation requires meeting requirements related to production reproducibility, chemical characterization, purity, batch-to-batch consistency, pharmacokinetics, toxicology, and quality control [135]. The development of semisynthetic derivatives and rationally designed analogs therefore represents a particularly attractive strategy for maintaining the antimycobacterial activity of natural products while improving their pharmacokinetic properties, selectivity, and tolerability. Overall, overcoming these limitations through structural optimization, development of semi-synthetic analogues, improved manufacturing processes and rigorous preclinical validation could facilitate the transformation of the most promising natural products into anti-TB candidates with improved efficacy, selectivity and safety [136].

## 6. Current Limitations

Although this review reports numerous studies on the antimycobacterial activity of natural products derived from microorganisms, it is important to acknowledge its limitations. The reported evidence is based primarily on *in vitro* studies; therefore, the *in vivo* application of natural products remains uncertain. They may not exhibit the same activity in preclinical or clinical settings due to pharmacokinetic barriers, limited bioavailability, toxicity, and the complexity of host–pathogen interactions. For many compounds investigated, the mechanism of action has not been elucidated, limiting the optimization of the antimycobacterial agent and its development as a drug. Another significant limitation is that most of the reported evidence has exploited standard bacterial strains, not multi-susceptible, MDR, and XDR clinical isolates. Therefore, the reported activities may not accurately reflect the antimycobacterial potential of these compounds against clinically relevant bacterial strains. Furthermore, variability in compound purity, based on isolation methods, represents another source of heterogeneity, influencing reported antimycobacterial activity and complicating comparisons with other studies. Despite these limitations, available evidence highlights microorganisms as a valuable source of structurally diverse natural products with promising antimycobacterial activity.

## Figures and Tables

**Table 1:** Summary of genome features, DNA replication, DNA repair, and transcription mechanisms in MTBC, focusing on differences from *E. coli* and associated antimicrobial drugs.

Process/Section	Components and Enzymes Involved	Main Differences Compared to *E. coli*	Associated Drugs
Genome Characteristics	~4000 genes (~3900–3950 protein-coding, 45–50 tRNAs, 3–4 rRNA operons, sRNAs). Size: ~4.4 Mb. GC content: 65%.	Predominantly clonal evolution, without horizontal gene transfer. High environmental and host resistance.	No antibiotics (represent a potential target for novel drug discovery).
DNA Replication	Replisome: DnaA-ATP, DnaB helicase, DnaG primase, beta-clamp, clamp-loading complex, core polymerase (alpha, epsilon).	The DnaC helicase loader and the chi/psi/gamma subunits are missing; the replisome is more compact. The theta subunit, which influences proofreading stabilization, is missing.	No antibiotics (represent a potential target for novel drug discovery).
DNA Repair	Mismatch repair: NucS system; BER enzymes; NER: UvrABC complex with UvrD helicase.	Lacks classical *E. coli* mismatch repair; relies on NucS; higher mutation rate when disrupted.	No current drugs targeting repair systems (emerging antimicrobial targets).
DNA Transcription	RNA polymerase (α2ββ′ω); sigma factors (σA, SigH, SigE, etc.).	Uses alternative sigma factor switching for stress/latency adaptation.	Rifampicin 10–20 mg/kg daily; targets β-subunit, inhibits mRNA synthesis.

**Table 2:** Structural and functional adaptations of the MTBC ribosome associated with latency.

Ribosomal Feature in MTBC	Molecular Mechanism/Functional Role	Impact on MTBC Persistence and Survival
B9 bridge between 23S rRNA and bS6 protein	Strengthens ribosome architecture and prevents premature dissociation of the 50S and 30S subunits during intracellular stress	Maintains ribosome integrity and supports long-term bacterial survival during latency
Additional rRNA loop conformations	Provide specific binding platforms for regulatory factors that modulate translation dynamics	Allow for fine-tuning of protein synthesis according to environmental conditions
Absence of bS21 protein in the small subunit	Reduces reliance on Shine–Dalgarno-mediated translation initiation and facilitates efficient recognition of leaderless mRNAs	Enables selective translation during persistence, when leaderless mRNAs become enriched
Presence of bL37 protein in the large subunit	Enhances peptidyl transferase center (PTC) efficiency and preserves translational activity under unfavorable conditions	Supports synthesis of essential proteins during nutrient limitation and cellular stress
Modified bS22 protein near the decoding center	Alters the structural configuration of the 30S decoding region and affects ribosome–antibiotic interactions	May contribute to altered antibiotic susceptibility and adaptation to antimicrobial pressure
C+ to C− ribosomal protein replacement during zinc limitation	Replaces zinc-binding ribosomal proteins with zinc-independent isoforms, allowing for zinc redistribution to essential cellular processes	Promotes metabolic adaptation and survival during host-induced nutritional stress
MPY-mediated ribosome hibernation	MPY binds the ribosomal A and P sites, reducing translation activity and protecting ribosomes from degradation and drug targeting	Preserves ribosomal pools during dormancy and enables rapid reactivation when favorable conditions return

**Table 3:** Antimycobacterial non-ribosomal peptide produced by Actinomycetes.

Compound	Producing Actinomycete	Structural Features	Target/ Mechanism	Tested Strains	Activity
Pyridomycin	*Streptomyces* sp. strain W3009	Cyclodepsipeptide	InhA inhibitor	*M. tuberculosis* H37Rv	IC_50_ 1.08 µM
Pyridomycin derivatives (E–F)	*Streptomyces* sp. strain W3009	Linear/cyclic analogues	InhA inhibitor	*M. tuberculosis* H37Rv	IC_50_ 6.9–9.14 µM
Actinomycin C1–C3	*S. pratensis*	Cyclic chromopeptides	DNA binding	*M. tuberculosis* H37Rv	MIC 0.039–0.0625 µg/mL
Actinomycin D	*S. parvus*	Chromopeptide	DNA intercalation	*M. tuberculosis* H37Rv	MIC 0.78 µg/mL
Actinomycin-X2	*S. smyrnaeus*	Actinomycin analogue	PknB, predicted by *in silico* analysis	*M. tuberculosis* H37Ra and H37Rv; *M. bovis*	MIC 1.56–2.64 µg/mL
Cyclomarin A	*Streptomyces* sp. strain CNB-982	Cyclic heptapeptide	ClpC1	*M. tuberculosis* H37Rv; clinical isolate XDR	MIC 0.5 µg/mL
Echinomycin	*S. fuscichromogenes*	Cyclic depsipeptide	DNA intercalator	*M. bovis*, *M. tuberculosis* H37Rv	MIC 0.1/0.5 µg/mL
Rufomycins	*S.* *atratus*	Cyclic heptapeptides	ClpC1	*M. tuberculosis* H37Rv	MIC 0.02–1.7 µM
Ilamycins	*S.* *atratus*	Cyclic heptapeptides	ClpC1	*M. tuberculosis* H37Rv	MIC 0.0096–10 µM
Atrovimycin	*S.* *atrovirens*	Cyclodepsipeptide	Unknown	*M. tuberculosis* H37Rv	MIC 1.88 µM
Ohmyungsamycins A/B	*Streptomyces* sp. strain SNJ042	Cyclic peptides	Host-directed: AMPK-dependent autophagy induction	*M. tuberculosis* H37Rv	IC_50_ 0.06–0.12 µM
Atratumycin	*S.* *atratus*	Cyclic decapeptide	Unknown	*M. tuberculosis* H37Ra and H37Rv	MIC 3.8–14.6 µM
Ecumicin	*Nonomuraea* sp.	Macrolactam peptide	ClpC1	*M. tuberculosis* H37Rv; Clinical isolates	MIC 0.34–1.5 µM
Taeanamide A	*Streptomyces* sp. strain AMD43	Cyclic lipopeptide	Unknown	*M. tuberculosis* mc^2^6230	MIC_50_ 27 µM
Taeanamide B	*Streptomyces* sp. strain AMD43	Linear lipopeptide	Unknown	*M. tuberculosis* mc^2^6230	MIC_50_ 63 µM
Thiolopyrrolone A	*Streptomyces* sp. strain BTBU20218885	Dithiolopyrrolone	Unknown	*M. bovis*, *M. tuberculosis* H37Rv	MIC 10 µM
Thiolutin	*Streptomyces* sp. strain BTBU20218885	Dithiolopyrrolone	Unknown	*M. bovis*, *M. tuberculosis* H37Rv	MIC 0.3125–0.625 µM

**Table 4:** Antimycobacterial Actinomycete-derived polyketides.

Compound	Producing Actinomycete	Structural Features	Target/ Mechanism	Tested Strains	Activity
Niphimycins C–E, 17-O-methylniphimycin	*Streptomyces* sp. strain IMB7-145	Alkylguanidyl-substituted polyketides	Membrane disruption	*M. tuberculosis* clinical isolates	MIC 4–32 µg/mL
Lincolnenin A	*S. lincolnensis*	Isomeric bianthracene	DNA intercalation	*M. tuberculosis* H37Ra	MIC 0.9 µM
Boromycin	*S. antibioticus*	Boron-containing polyether macrolide	Membrane potential collapse, ATP depletion	*M. tuberculosis* H37Rv, BCG	MIC 0.2 µM
Steffimycins	*Streptomyces* sp. strain OPMA02852	Anthracycline-like polyketides	Redox/DNA interference	*M. bovis* BCG	MIC 1.4–21.7 µM
Diazaquinomycins A,J,H	*M. maritima* strain B026	Diazaanthraquinone core	Redox imbalance	*M. tuberculosis*, MDR strains	MIC 0.10–0.34 µM
Murayaquinone	*Streptomyces* sp. strain TBRC7642	Quinone polyketide	ROS generation	*M. tuberculosis* H37Ra	MIC 9.65 µM
Furaquinocin D	*Streptomyces* sp. strain TBRC7642	Quinone polyketide	Oxidative stress	*M. tuberculosis* H37Ra	MIC 129.40 µM
Dinactin	*S. puniceus*	Polyether macrotetrolide	Ion transport disruption	*M. tuberculosis* H37Rv	MIC 1 µg/mL
Treponemycin	*S. mutabilis*	18-membered macrolide	Threonyl-tRNA synthesis inhibition	*M. tuberculosis*	MIC 13.3 µg/mL
Desertomycin A, Desertomycin 44-1, Desertomycin 44-2	*S. flavofungini* strain TRM90047	Polyether macrolides with a macrocyclic lactone ring, multiple ether bonds, high stereochemical complexity	Multi-target activity on protein synthesis proteins (RpsL, RplC) and on the ClpC1 protein control system.	*M. tuberculosis*	50% growth inhibition at 25–50 µg/mL
Desertomycin G	*S. althioticus* strain *MSM3*	Polyether macrolides with a macrocyclic lactone ring, multiple ether bonds, high stereochemical complexity	Undefined	*M. tuberculosis* H37Rv, MDR strains;	MIC 16 µM
Azalomycin B	*Streptomyces* sp. strain BCC71188	Macrodiolide polyether	Membrane disruption	*M. tuberculosis* H37Ra	MIC 0.78 µg/mL
Chrysomycin A	*Streptomyces* sp. strain OA161	Angucycline polyketide	DNA damage	*M. tuberculosis* H37Ra	MIC 3.125 µg/mL
Frenolicins A,G	*Streptomyces* sp. strain TBRC17107	Naphthoquinone polyketide	Redox cycling	*M. tuberculosis* H37Ra	MIC 25 µg/mL
Kimidinomycin	*Streptomyces* sp. strain KKTA-0263	Aromatic polyketide	Not defined	*M. bovis* BCG	MIC 23.66 µg/mL
Nybomycin	*Streptomyces* sp. strain MS44	Aromatic naphthoquinone; planar highly conjugated polycyclic core with quinone carbonyl groups conferring redox activity	Interference with DNA structure	*M. bovis* BCG	MIC 1.0 µg/mL
Aranciamycins (I, J, A)	*Streptomyces* sp. strain CMB-M0150	Anthracyclines characterized by a planar aromatic tetracyclic anthraquinone nucleus containing quinone carbonyl groups involved in redox activity	Undefined	*M. bovis*	MIC 10 μM

**Table 5:** Other actinomycete-derived compounds with antimycobacterial activity.

Compound	Producing Actinomycete	Structural Features	Target/Mechanism	Tested Strains	Activity
Mavintramycins A–G, amycetin, plicacetin	*Streptomyces* sp. strain OPMA4055	Nucleosides modified with core cytosine + amosamine + amicetose; variable substituent R	Inhibition of protein synthesis by binding to 23S rRNA	*M. bovis BCG*	MIC: 0.63–13.01 µg/mL
(2S,2″S)-6-lavandulyl-7,4′-dimethoxy-5,2′-dihydroxyflavanone	*Streptomyces* sp. strain G248	C6–C3–C6 polyoxygenated flavanone with lavandularyl substituent and OMe/OH groups	Undefined	*M. tuberculosis* H37Rv	90% reduction to 48 µg/mL
2,4-Di-tert-butylphenol	*S. bacillaris* strain ANS2	Alkyl-substituted phenol (2,4-di-tert-butyl-phenol)	Binding a lysine aminotransferase (docking)	*M. tuberculosis* H37Rv, MDR clinical strain	MIC: 48.47 µg/mL; 242.37 µg/mL
Lassomycin	*L. kentuckyensis*	Cyclic macrolactam ring with a threaded C-terminal peptide chain	Targets ClpC1 ATPase	*M. tuberculosis* H37Rv; clinical isolates resistant to INH, RIF, STR, EMB, PZA, FQ; mc^2^6020 strain	MIC 0.78–1.56 μg/mL (H37Rv); 0.78–3.1 μg/mL (drug-resistant strains); 0.39–0.78 μg/mL (mc^2^6020)
Streptomycobactin	*Streptomyces*	Linear peptide siderophore composed of 20 amino acids	Undefined	*M. tuberculosis* mc^2^6020, H37Rv	MIC 0.03 µg/mL
Amycobactin	Amycolatopsis	Peptide siderophore with cyclic macrolactone structure	Target identified as SecY	*M. tuberculosis* mc^2^6020, H37Rv	MIC 4–8 µg/mL
Kitamycobactin	Kitasatospora	Looped peptide siderophore	Targets the essential ClpP1P2C1 system	*M. tuberculosis* mc^2^6020, H37Rv	MIC 0.06 µg/mL
Marfomycin D	*Streptomyces*	Non-ribosomal peptide	Undefined	*M. tuberculosis* mc^2^6020, H37Rv	MIC 1 µg/mL for mc^2^6020 and 0.03 µg/mL for H37Rv

**Table 6:** Other bacterial-derived compounds with antimycobacterial activity.

Compound	Producing Actinomycete	Structural Features	Target/Mechanism	Tested Strains	Activity
Teixobactin	*E. terrae*	Non-ribosomal lipo-depsipeptide containing non-canonical amino acids and a lipid moiety	Binds essential lipid intermediates involved in cell-wall precursor transport, including those required for peptidoglycan and arabinogalactan biosynthesis	*M. tuberculosis* H37Rv	MIC: 0.125 µg/mL
Evybactin	*P. noenieputensis* strain DSM 25462	Cyclic depsipeptide composed of 11 amino acid residues, including N-methylhistidine, N-formylated tryptophan and β-aspartate residues	Targets DNA gyrase A	*M. tuberculosis* H37Rv, mc^2^6020	MIC 0.25 µg/mL

**Table 7:** Fungal-derived compounds with antimycobacterial activity.

Compound	Producing Fungus	Structural Features	Target/Mechanism	Tested Strains	Activity
Gliotoxin	*Aspergillus* sp. strain SCSIO	Epipolythiodioxopiperazine (ETP); cyclic diketopiperazine core containing an intracyclic disulfide bridge	Undefined	*M. tuberculosis* H37Rv	MIC_50_ < 0.03 µM
12,13-Dihydroxyfumitremorgine C	*Aspergillus* sp. strain SCSIO	Prenylated indole alkaloid; indole nucleus fused with diketopiperazine ring and hydroxyl substituents	Undefined	*M. tuberculosis* H37Rv	MIC_50_ 2.41 µM
Helvolic acid	*Aspergillus* sp. strain SCSIO	Fusidane-type steroidal triterpenoid with oxygenated pentacyclic skeleton	Undefined	*M. tuberculosis* H37Rv	MIC_50_ 0.894 µM
Emodin	*P. citrinum*	Anthraquinone derivative; aromatic system with carbonyl and hydroxyl groups	Undefined	*M. bovis* BCG; *M. tuberculosis* H37Rv, M442, M299	MIC_50_ 0.4 µM against BCG; 5.0–8.2 µM against *M. tuberculosis* strains
Mycoleptone A	*M. indicus*	Fungal polyketide with oxygenated aromatic structure	Undefined	*M. bovis* BCG; *M. tuberculosis* H37Rv, M442, M299	MIC_50_ 1.7 µM against BCG; 9.2–37.4 µM against *M. tuberculosis* strains
Austdiol	*M. indicus*	Oxygenated aromatic polyketide	Undefined	*M. bovis* BCG; *M. tuberculosis* H37Rv, M442, M299	MIC_50_ 11.7 µM against BCG; 10.8–33.4 µM against *M. tuberculosis* strains
Eugenitin	*M. indicus*	Quinone derivative with condensed aromatic system	Undefined	*M. bovis* BCG; *M. tuberculosis* H37Rv, M442, M299	Weak activity; MIC_50_ 79.1 µM (H37Rv), 81.2 µM (M442), >100 µM (M299)
δ-Lactam derivative	*H. grisea*	Lactam-containing fungal metabolite	Undefined	*M. bovis* BCG; *M. tuberculosis* H37Rv, M442, M299	MIC_50_ 20.5 µM against BCG and >100 µM against *M. tuberculosis* strains
(R)-(+)-Lasiodiplodin	*S. tamaensis*	12-membered macrolactone ring linked to oxygenated resorcinol aromatic moiety; R-(+) chiral center	Inhibits class II fructose-1,6-bisphosphate aldolase	*M. bovis* BCG; *M. tuberculosis* H37Rv and M299	MIC_50_ 6.7 µg/mL (BCG), 60.4 µg/mL (H37Rv), 92.2 µg/mL (M299).
(24E)-7α-Hydroxy-3-oxolanosta-8,24-dien-26-oic acid	*G. orbiforme*	Lanostane triterpenoid; C30 tetracyclic skeleton with hydroxylated and oxygenated groups	Undefined	*M. tuberculosis* H37Ra	MIC 3.13 µg/mL
(24E)-3β,15α-Diacetoxylanosta-7,9(11),24-trien-26-oic acid	*G. orbiforme*	Lanostane triterpenoid with acetoxy substitutions	Undefined	*M. tuberculosis* H37Ra	MIC 0.391 µg/mL
Ganorbiformin C	*G. orbiforme*	Lanostane triterpenoid derivative	Undefined	*M. tuberculosis* H37Ra	MIC 1.56 µg/mL
(24E)-3β,15α-Diacetoxy-7α-hydroxylanosta-8,24-dien-26-oic acid	*Ganoderma* sp. strain BCC 16642	Oxygenated lanostane triterpenoid	Undefined	*M. tuberculosis* H37Ra	MIC 1.56 µg/mL
3β,15α-Diacetoxylanosta-8,24-dien-26-oic acid	*Ganoderma* sp. strain BCC 16642	Lanostane triterpenoid with acetoxy groups	Undefined	*M. tuberculosis* H37Ra	MIC 0.781 µg/mL

## Data Availability

No new data were created or analyzed in this study. Data sharing is not applicable to this article.
